# Associations of Diet and Physical Activity with Risk for Gestational Diabetes Mellitus: A Systematic Review and Meta-Analysis

**DOI:** 10.3390/nu10060698

**Published:** 2018-05-30

**Authors:** Jovana Mijatovic-Vukas, Louise Capling, Sonia Cheng, Emmanuel Stamatakis, Jimmy Louie, N. Wah Cheung, Tania Markovic, Glynis Ross, Alistair Senior, Jennie C. Brand-Miller, Victoria M. Flood

**Affiliations:** 1 Charles Perkins Centre, The University of Sydney, Sydney 2006, Australia; jmij1646@uni.sydney.edu.au (J.M.-V.); emmanuel.stamatakis@sydney.edu.au (E.S.); jimmyl@hku.hk (J.L.); wah.cheung@sydney.edu.au (N.W.C.); tania.markovic@sydney.edu.au (T.M.); alistair.senior@sydney.edu.au (A.S.); jennie.brandmiller@sydney.edu.au (J.C.B.-M.); 2 The School of Life and Environmental Sciences, The University of Sydney, Sydney 2006, Australia; 3 Faculty of Health Sciences, The University of Sydney, Lidcombe 2141, Australia; acap7726@uni.sydney.edu.au (L.C.); sonia.cheng@sydney.edu.au (S.C.); 4 Prevention Research Collaboration, School of Public Health, The University of Sydney, Sydney 2006, Australia; 5 School of Biological Sciences, Faculty of Science, The University of Hong Kong, Pokfulam, Hong Kong, China; 6 Westmead Hospital, Western Sydney Local Health District, Westmead 2145, Australia; 7 Westmead Clinical School, Faculty of Medicine and Health, The University of Sydney, Westmead 2145, Australia; 8 Department of Diabetes and Endocrinology, Westmead Hospital, Westmead 2145, Australia; 9 Royal Prince Alfred Hospital, Sydney Local Health District, Camperdown 2050, Australia; glynis.ross@health.nsw.gov.au; 10 Central Clinical School, Faculty of Medicine and Health, The University of Sydney, Sydney 2006, Australia; 11 School of Mathematics and Statistics, The University of Sydney, Sydney 2006, Australia

**Keywords:** pregnancy, pre-pregnancy, gestational diabetes, diet, physical activity, exercise

## Abstract

Rising rates of gestational diabetes mellitus (GDM) and related complications have prompted calls to identify potentially modifiable risk factors that are associated with gestational diabetes mellitus (GDM). We systematically reviewed the scientific literature for observational studies examining specific dietary and/or physical activity (PA) factors and risk of GDM. Our search included PubMed, Medline, CINAHL/EBSCO, Science Direct and EMBASE, and identified 1167 articles, of which 40 met our inclusion criteria (e.g., singleton pregnancy, reported diet or PA data during pre-pregnancy/early pregnancy and GDM as an outcome measure). Studies were assessed for quality using a modified Quality Criteria Checklist from American Dietetic Association. Of the final 40 studies, 72% obtained a positive quality rating and 28% were rated neutral. The final analysis incorporated data on 30,871 pregnant women. Dietary studies were categorised into either caffeine, carbohydrate, fat, protein, calcium, fast food and recognized dietary patterns. Diets such as Mediterranean Diet (MedDiet), Dietary Approaches to Stop Hypertension (DASH) diet and Alternate Healthy Eating Index diet (AHEI) were associated with 15–38% reduced relative risk of GDM. In contrast, frequent consumption of potato, meat/processed meats, and protein (% energy) derived from animal sources was associated with an increased risk of GDM. Compared to no PA, any pre-pregnancy or early pregnancy PA was associated with 30% and 21% reduced odds of GDM, respectively. Engaging in >90 min/week of leisure time PA before pregnancy was associated with 46% decreased odds of GDM. We conclude that diets resembling MedDiet/DASH diet as well as higher PA levels before or in early pregnancy were associated with lower risks or odds of GDM respectively. The systematic review was registered at PROSPERO (www.crd.york.ac.uk/PROSPERO) as CRD42016027795.

## 1. Introduction

Gestational diabetes mellitus (GDM) is defined as diabetes or glucose intolerance occurring for the first time in pregnancy [1]. Although diagnostic criteria vary, GDM affects approximately 15–20% [2] of pregnancies, reaching 30% [3,4] in some parts of the world. The World Health Organization’s (WHO) new diagnostic criteria explain some, but not all, of the recent rise in prevalence [4]. Delayed age of motherhood, obesity and migration of higher risk population groups to regions of lower risk also contribute to increasing prevalence of GDM [5]. Women with GDM have up to 70% chance of progressing to type 2 diabetes mellitus (T2DM) within 28 years post-partum [6]. GDM also increases the risk of macrosomia, hypoglycemia [7] and epigenetic changes in the infant, resulting in a new generation susceptible to obesity and type 2 diabetes later in life [8].

Current treatment options include changes in lifestyle, e.g., more physical activity (PA) and improved diet quality through Medical Nutrition Therapy (MNT) [9]. MNT aims to achieve and maintain euglycemia through consumption of appropriate meal portions, distribution of carbohydrates [10] and consumption of foods with a lower glycemic index (GI) [4,9]. A recently published Cochrane review and meta-analysis of randomized controlled trials (RCT) demonstrated that provision of more intensive health care such as additional dietary counseling and exercise, lead to an improved glycated hemoglobin (HbA1c), lower incidence of large-for-gestational-age (LGA) infants, decreased weight gain in pregnancy and lower rate of depression in mothers three months post-partum, compared to standard care [9]. However, when lifestyle approaches alone are not sufficient, insulin or other anti-hyperglycemic pharmacological therapies are often prescribed [4,9].

The increasing prevalence of GDM has prompted calls to identify key lifestyle factors that either prevent or promote onset of the disease. The Finnish Gestational Diabetes Prevention Study (RADIEL) study suggested that the risk of GDM can be reduced by approximately 40% by following a physically active lifestyle and a diet enriched with fruits, vegetables and wholegrain cereals as per Nordic Nutrition Recommendations [11]. While higher fruit and vegetable intake has also been reported to reduce all-cause mortality, particularly cardiovascular mortality in a general population [12], other dietary patterns, habits and components may be relevant risk factors for GDM.

PA has long been prescribed to patients with diabetes due to improvements in glycemia and insulin sensitivity [13]. It has been proposed that PA achieves these benefits by promoting an increase in skeletal muscle glucose uptake in resistance training or increase mitochondrial density and expression of glucose transporter proteins that are evident in aerobic exercises [13,14]. Women are currently advised to take part in 150–300 min/week in moderate-intensity aerobic PA both during pregnancy and after delivery [15] as there is a strong inverse association between PA and excessive weight gain in pregnancy, postpartum depression as well as GDM [16]. However, despite the benefits, only 23–29% of pregnant women meet the recommendations [16]. Women generally decrease their incidental PA as pregnancy progresses [17] and spend the majority of their day being sedentary (up to 60%), as shown by motion sensor data from the US [18].

The aim of the present study was to undertake a systematic literature search of observational studies investigating the associations between diet and PA aspects before and in early pregnancy that are associated with risk of GDM. In addition, we conducted a meta-analysis on PA studies to examine the associations of specific types or duration of PA with GDM risk.

## 2. Materials and Methods

### 2.1. Eligibility Criteria

Longitudinal and cohort studies containing information on diet and PA either prior to or at the commencement of a singleton human pregnancy were included. Although GDM was the main outcome measure, studies that reported other outcomes (e.g., pre-eclampsia, macrosomia and large/small for gestational age offspring) were also included. Studies were excluded if they were published in a language other than English, subjects had diabetes mellitus in pregnancy diagnosed at the start of the pregnancy, or an underlying medical condition that affected digestion and absorption of nutrients (e.g., sleeve gastrectomy or gastric bypass surgery).

### 2.2. Information Sources and Search

This systematic review was registered at PROSPERO (www.crd.york.ac.uk/PROSPERO) as CRD42016027795. A systematic literature search was undertaken initially 22 October 2015 and was later updated 2 February 2017 by three independent researchers (J.M-V., L.C. and S.C.). The search was conducted using Medline, PubMed, Science Direct, EMBASE and Cumulative Index to Nursing and Allied Health Literature (CINAHL) databases with interest in studies that reported on the relationship between diet and/or PA during pre-pregnancy or early pregnancy and risk of GDM. Additionally, we hand searched reference lists to obtain more studies. For a complete list of search terms, please refer to Table S1. A time frame 1985–2017 was selected as it reflected the current context of high prevalence of GDM as well as to capture good quality cohort and longitudinal studies.

### 2.3. Quality Assessment and Data Extraction

To determine study quality, studies were assessed independently (J.M-V., L.C., S.C.) using a modified version of the Quality Criteria Checklist found in the American Dietetic Association (ADA) Evidence Manual [19]. Information about confounding variables and adjusted data was also collected from included studies to highlight study strengths or weaknesses. We assigned either a positive, neutral or a negative value for studies that were of excellent, good and poor quality respectively. Study characteristics were extracted into a pre-determined table that collected information including author, year of publication, participant number, study selection criteria, data collection methods, GDM diagnostic criteria as well as results and how they were deduced. Data were cross-checked by co-author (VF) for any errors and discrepancies.

### 2.4. Statistical Analysis

Our primary outcome measure was onset of GDM. We quantified study results using odds ratios (OR), which along with their sample variances, were calculated using the *escalc* function in *metafor* R package (Maastricht University, Maastricht, Netherlands) [20]. A positive effect size suggests that the odds of GDM are higher in group A (active) than B (inactive). Where results were reported as stratified across groups a combined effect-size was calculated following Borenstein and colleagues [21]. Where results were reported in text as relative risk (RR), we used the following equation (Equation 1) from Deeks and Altman [22] to convert values reported to OR, where *p_c_* is the typical event rate without treatment:

(1) $$\mathrm{OR}\;=\frac{\mathrm{RR}\;\left(1\;-p_{c}\right)}{1\;-p_{c}\mathrm{RR}}$$

For analysis, odds ratios were log transformed (i.e., lnOR), and in places we back-transform overall lnOR for interpretation by raising *e* (exponential) to the power of the lnOR.

We analyzed effect sizes using a random-effects meta-analysis (REMA) model, implemented in the R package *metafor.* Statistical heterogeneity was quantified via the heterogeneity statistic, *I*^2^—a type of intra-class correlation [23]. *I*^2^ corresponds to the percentage of among effect sizes variance, that cannot be attributed to sampling. An *I*^2^ value determined variability of results between different studies as either low (25%), moderate (50%) or high (75%). We were unable to perform a meta-analysis on dietary studies as they were too diverse in the aspects of the diet they report on. Therefore, we report on meta-analyses conducted on PA studies only.

We assessed the risk of publication bias across studies by developing a funnel plot with lnOR scale and inverse standard error as x and y-axis respectively. We also used a *regtest* function in *metafor* to determine funnel plot asymmetry. It should be noted that tests of funnel plot asymmetry can be unreliable indicators of publication bias where the number of effect sizes is small (<10) and/or there is substantial heterogeneity. Thus, in the main text, we only present and interpret results from publication bias tests where the number of effect sizes was greater than 10. Statistical significance of the overall effect of PA was inferred when the *p*-value was <0.05 and 95% Confidence Intervals (CI) did not contain 0.

## 3. Results

### 3.1. Studies Identified

**Figure 1:**
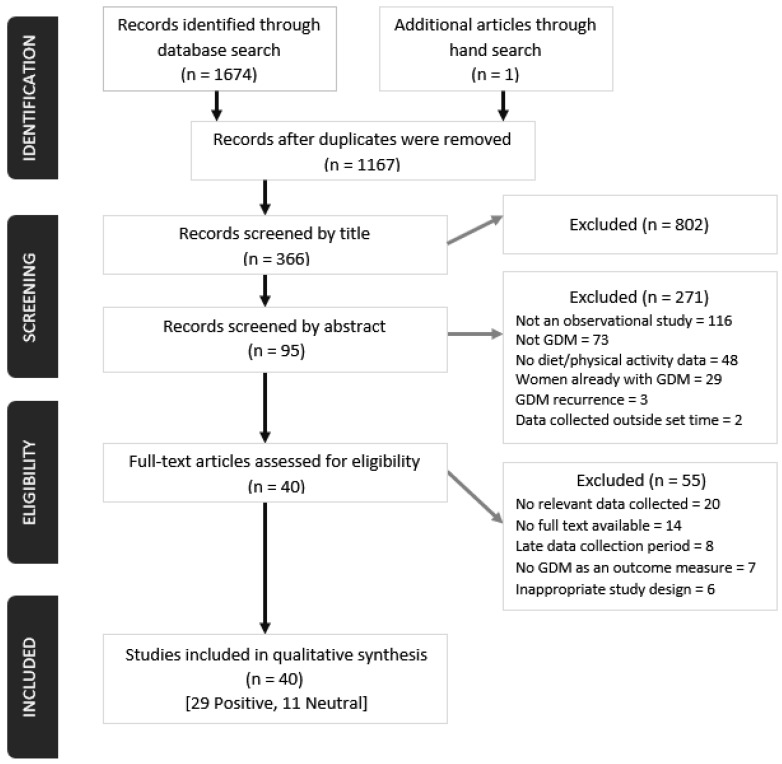
PRISMA flow diagram of screening, selection process and inclusion of studies.

We extracted 1166 articles from the database searches, and one article was identified through hand-search. After screening and assessing for eligibility, we identified 40 journal articles which met inclusion criteria (Figure 1). Of these 40 articles, 23 reported data only on dietary intake, 15 only on PA and two articles reported on both dietary intake and PA. Twenty-nine studies (72%) obtained a positive quality rating and 11 (28%) were rated neutral (Table S2). All the articles reported findings from prospective cohort studies of which there were multiple publications from four major studies including Nurses’ Health Study II (*n* = 14) [24,25,26,27,28,29,30,31,32,33,34,35,36,37], Omega (*n* = 7) [38,39,40,41,42,43,44], Australian Longitudinal Study on Women’s Health (*n* = 4) [45,46,47,48] and Project Viva (*n* = 2) [49,50]. The most common reasons for exclusion of publications were unavailability of full texts, no relevant data collected necessary for the present review and late recruitment of study participants, consequently capturing dietary and PA information that were not reflective of the pre-pregnancy or early pregnancy period.

### 3.2. General Characteristics of Studies

The review captured data on 30,871 pregnancies of which 1980 (7%) developed GDM. The studies provided information on women from multiple populations including 26 American [24,25,26,27,28,29,30,31,32,33,34,35,36,37,38,39,40,41,42,43,44,48,49,51,52,53], five Australian [45,46,47,48,54], two Hispanic American [55,56] and one each for the following: Iranian [57], Danish [58], Canadian [59], Pakistani [60], Norwegian [61], Spanish [62], and multi-centre Mediterranean Study (Algeria, France, Greece, Italy, Lebanon, Malta, Morocco, Serbia, Syria and Tunisia) [63], The number of participants per study ranged from 97 to 71,239 and were published between 1997–2016, with an age range of 16–48 years as reported in 27 studies. Of 22 studies that reported retention rate, 14 had ≥80% [38,40,44,47,49,50,54,56,58,60,61,62,63], 7 had 50–79% [41,42,43,48,51,55,59], and only one <50% [47].

The reported GDM diagnostic methods included a 100 g (*n* = 12) [38,40,41,42,43,49,50,53,55,56,57,60], 75 g (*n* = 5) [47,48,54,61,63], 50 g (*n* = 1) [51], a combination of these (*n* = 2) [59,62] oral glucose tolerance tests (OGTT) or were extrapolated from medical records (*n* = 20) [24,25,26,27,28,29,30,31,32,33,34,35,36,37,39,44,45,46,52,58,64]. Multiple diagnostic criteria were used to ascertain GDM status and included (1) 1997 American Diabetes Association (ADA) criteria (*n* = 2; fasting ≥ 105 mg·dL^−1^, 1 h ≥ 190 mg·dL^−1^, 2 h ≥ 165 mg·dL^−1^, ≥145 mg·dL^−1^) [39,40], (2) 2004 ADA criteria (*n* = 10; fasting ≥ 95 mg·dL^−1^, 1 h ≥ 180 mg·dL^−1^, 2 h ≥ 155 mg·dL^−1^ ≥ 140 mg·dL^−1^) [41,42,43,49,50,55,56,57,60,62], (3) 2010 International Association of the Diabetes and Pregnancy Study Group (IADPSG) criteria (*n* = 3; fasting ≥ 5.1 mmol·L^−1^, 1 h ≥ 10.0 mmol·L^−1^, ≥ 8.5 mmol·L^−1^) [54,61,63], (4) 1998 Australasian Diabetes in Pregnancy Society (ADIPS) criteria (*n* = 3; fasting ≥ 5.6 mmol·L^−1^ and/or 2-h ≥ 8.0 mmol·L^−1^) [46,47,48] or were not reported (*n* = 22) [24,25,26,27,28,29,30,31,32,33,34,35,36,37,38,44,45,51,52,53,58,59]. Thirty-six studies reported on pre-pregnancy body mass index (BMI), of which 20 were within the normal range [24,25,26,27,28,29,30,33,34,35,36,39,41,42,43,49,57,61,62], one overweight [51], one obese [54] and 14 were categorised into multiple groups [32,37,40,47,48,50,52,53,55,56,58,59,60,63] rather than providing an overall average. Twenty-two publications reported data from the pre-pregnancy period [24,26,27,28,29,30,31,32,33,34,35,36,37,38,41,42,45,46,47,51,62], ten focused on early pregnancy [39,48,50,52,54,57,58,60,63], seven on both [40,43,49,55,56,59,61] and one was unclear [44]. Refer to Table S3 for more information about study characteristics.

### 3.3. Diet Related Studies

**Figure 2:**
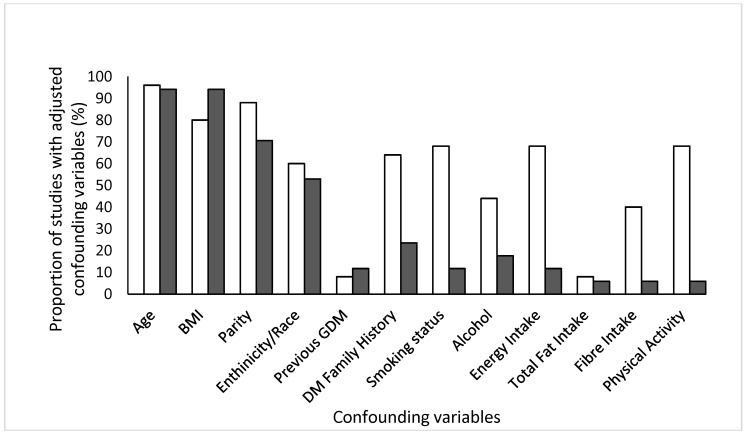
Confounding variables that were adjusted for in studies collecting information on dietary intake (white bars) and physical activity levels (black bars). Age, BMI and parity were most commonly adjusted confounding variables in observational studies reporting on either diet or physical activity.

To account for the diversity of 25 dietary studies identified after inclusion, we categorised them into one of seven themes: carbohydrates, fat intake, protein, fast food intake, caffeine, calcium intake and commonly recognised dietary patterns. The predominating dietary collection method was a validated Food Frequency Questionnaire (FFQ) (*n* = 23) [24,25,26,27,28,29,30,31,33,34,35,41,42,43,45,46,47,57,62,63]. The remaining two studies used a rapid food screener [51] and an interview [58] to collect dietary data. Studies focusing on early pregnancy collected dietary data <22 weeks into pregnancy. When studies reporting on diet and PA were compared with respect to adjusted confounding variables (Figure 2), we observed that age, BMI and parity were most common in both. Only 70% of diet related and 10% PA studies adjusted for energy intake.

#### 3.3.1. Carbohydrates (Fruit, Fiber, Beverages, Potato)

Five studies reported on high carbohydrate foods, including fruit, fiber, potato and beverage intake and their respective associations to GDM risk [26,27,30,31,34]. High pre-pregnancy fruit intake was not associated with an increase in GDM risk (RR high vs. low intake = 0.93, 95% CI: 0.76–1.16) [31], however fruit fiber [34] was reported to be protective (RR fruit fiber = 0.66, 95% CI: 0.51–0.86). Although high compared to low apple intake suggested non-significant protection from GDM risk (RR apple = 0.81, 95% CI: 0.65–1.01), the overall trend across quintiles of apple consumption reached statistical significance (*p*-trend < 0.05) [31]. Protective effects were also evident when consumption of total dietary fiber and cereal fiber were examined (RR total fiber = 0.67, 95% CI: 0.51–0.90; RR cereal fiber = 0.76, 95% CI: 0.59–0.99) [34].

Higher frequency of potato intake increased the risk of GDM (RR high vs. low frequency intake = 1.62, 95% CI: 1.24–2.13) [27]. However, frequent consumers of potato tended to be current smokers, had higher BMI and lower diet quality as assessed by the Alternate Healthy Eating Index (AHEI) 2010 score [27]. In contrast, a study by Karamanos and colleagues found that women who went on to develop GDM consumed less potatoes and cereals than those that did not develop it [63]. Replacing two servings of potatoes per week for other vegetables types, legumes or wholegrain foods resulted in a 9%, 10% and 17% GDM risk reduction respectively [27]. No significant association was observed between potato crisps or corn chips and GDM risk after adjustment of confounding variables including age, parity, race, family history of diabetes, smoking, PA, energy intake, diet quality and BMI [27].

The relationship between 100% fruit juice consumption and GDM onset was nonlinear, with the lowest risk observed in women with moderate fruit juice intake [31]. In contrast, higher sugar sweetened beverage (SSB) intake was associated with GDM risk (RR ≥ 5 week = 1.23, 95% CI: 1.05–1.45, *p*-value = 0.005) [30]. When different sub-types of SSB were taken into account, the strongest association was observed for sugar sweetened cola (RR high vs. low intake = 1.29, 95% CI: 1.07–1.55) but not for non-cola SSB (RR high vs. low = 0.99, 95% CI: 0.78–1.25) [30].

#### 3.3.2. Fat Intake (i.e., Total, Monounsaturated Fatty Acids, Dietary Cholesterol, Egg Intake)

Higher intake of animal, cholesterol and monounsaturated fatty acids (MUFA) were significantly associated with increased risk of GDM [29]. When comparing highest to lowest quintile of animal fat intake (% E), the risk increased by ~90% (RR = 1.88, 95% CI: 1.36–2.60) [29]. Similarly, a comparison between the highest and lowest quintile of cholesterol intake elucidated a positive relationship with GDM risk (RR = 2.35, 95% CI = 1.35, 4.09). On the contrary, Baptise-Roberts and colleagues reported no association between either cholesterol or total fat intake with a high glucose response following a glucose challenge test [51].

While no associations were observed between total omega-3 or total omega-6 fatty acids and risk of GDM in one study [29], another noted that women who developed GDM had a lower n-6/n-3 ratio, a higher intake of n-3 fatty acid and polyunsaturated fats than their non-GDM counterparts [50]. Each 300 mg/day intake of alpha-linolenic acid, was associated with an increased risk for GDM, with an OR = 1.29 (95% CI: 1.04–1.60) [50]. Karamanos and colleagues reported that olive oil was consumed in higher quantities in women who went on to develop GDM compared to those that did not [63], however no association analysis was presented [63]. With regards to egg consumption, one study suggested that high intakes increased the risk of GDM by a 1.77-fold [42], while another found no such association [24].

#### 3.4.3. Protein Intake (i.e., Meat, Iron, Heme)

Bao and colleagues reported that an intake of protein from animal origin increased the risk of GDM by ~50%, whereas an intake of protein sourced from vegetables was protective by 30% [24]. Similarly, a low carbohydrate dietary pattern with high animal protein and animal content was associated with a 36% increase risk of GDM, whereas a low carbohydrate diet containing high intake of plant-sourced protein and fat was not associated with any increased risk [25]. Replacing 5% energy of animal protein for protein of plant origin reduced GDM risk by 51% [24].

Neither a high meat score before pregnancy calculated using The Rapid Food Screener [51] or a high red meat intake in early pregnancy [50] were able to predict GDM risk in the two studies. On the contrary, three studies concluded that women with a high pre-pregnancy red meat intake had between 1.4–2.0 times the risk of developing GDM [24,35,47]. There are similar inconsistent findings for processed meat intake and GDM risk. Two studies reported a statistically significant increased risk for GDM, ranging between 48–68% during the pre-pregnancy period [24,35], whereas the remaining study found no such association [50]. A positive relationship was observed between higher pre-pregnancy iron or heme intake and GDM [28,43]. While Behboudi-Gandevani and colleagues [57] observed no statistically significant differences in iron or zinc intake in women with or without GDM, women with GDM did have a statistically significant higher serum iron level in early pregnancy.

#### 3.3.4. Caffeine

Two studies [38,58] reported on caffeine intake and risk of GDM. Whilst both captured caffeine intake during the pre-pregnancy period, Hinckle and colleagues additionally looked at tea intake [58]. Coffee consumption was reported to have a protective affect against GDM in one study [RR = 0.48 (95% CI: 0.28–0.82)] [38], but failed to reach a statistical significance in the other (RR ≥ 8 vs. 0 cups/day = 0.89, 95% CI: 0.64–1.25) [58]. Consumption of decaffeinated coffee was not associated with risk reduction [38]. Increasing frequency of tea intake indicated a potential protective effect against GDM risk, albeit statistically insignificant (RR ≥ 8 vs. 0 cups/day = 0.77, 95% CI: 0.55–1.0) [58].

#### 3.3.5. Fast Food Intake

Increasing frequency of fast food intake prior to pregnancy was associated with a statistically significant increased risk [26] or incidence [62] of GDM. The reported RR for ≥7/week vs. <1/week = 2.18, 95% CI: 1.53–3.09) [26] and Odds Ratio (OR) for highest vs. lowest frequency intake = 1.86, 95% CI: 1.13–3.06) [62]. Women with greater fast food consumption were typically younger, current smokers, multiparous, less physically active and followed diets that were either less adherent to the Mediterranean Diet (MedDiet) pattern [62] or had an overall lower AHEI-2010 diet quality score [26].

#### 3.3.6. Calcium/Dairy Intake

Total pre-pregnancy dairy intake was not associated with risk of GDM [24,41]. Habitual maternal intake of low-fat dairy suggested a non-significant inverse association with GDM risk (RR highest vs. lowest quintile = 0.57, 95% CI: 0.32–1.02) [41], however the overall trend across quartiles of low-fat dairy intake reached statistical significance (*p*-trend < 0.05). Interestingly, Schoenacker and colleagues (2015) observed fruit and low-fat dairy as a dietary pattern, but found no association with GDM risk [47]. With respect to calcium intake, an inverse association with GDM risk was observed, albeit statistically insignificant [41]. When quintiles were grouped into higher vs. lower level of intake, women who consumed ≥795 mg Calcium/day had a 42% GDM risk reduction when compared to those who had <795 mg/day [41].

#### 3.3.7. Recognised Dietary Patterns

MedDiet was the most consistently reported protective dietary pattern against GDM risk, reaching statistical significant in all four studies [33,46,47,63]. In a comprehensive review by Radd-Vagenas and colleagues, MedDiet is defined as a diet containing higher bread, cereal, legume, vegetable, fruit, fish and olive oil intake and smaller or limited intake of animal fat, meat and eggs [65]. The extent of MedDiet protectiveness ranged from 15–38%. Although women with better MedDiet compliance had lower incidence of GDM than their non-compliant counterparts, Karamanos and colleagues reported that GDM incidence greatly differed when comparing ADA and IADPSG diagnostic criteria between the compliant groups (8% vs. 24%, respectively) [63].

Adherence to a diet with a high AHEI 2010 score was associated with a reduced risk of GDM by 19% [37] or 46% [33]. When additional lifestyle factors were taken into account such as regular PA, normal BMI, non-smoker, the association with risk reduction was 83% [37]. Similarly to the AHEI scoring system, some studies used an Australian Recommended Food Score (ARFS) [45] or Dietary Approaches to Stop Hypertension (DASH) score [33]. A high ARFS was not associated with GDM risk [45], whereas a greater DASH diet compliance was associated with a 34% GDM risk reduction [33]. With respect to Prudent and Western diets, there were some conflicting results. Whilst compliance to a Prudent or Western diet resulted in a negative and positive association with GDM risk respectively in one study [35], a second study observed no such relationships [50].

### 3.4. Physical Activity

The relationship between PA and risk of GDM was examined by 17 publications, including the Nurse’s Health Study II (NHS II, *n* = 3), OMEGA Study (*n* = 3), Projecta VIVA (*n* = 1), the Australian Longitudinal Study on Women’s Health (*n* = 1). Data collection methods included interviews (*n* = 2), questionnaires (*n* = 14, of which 12 were validated) and a self-report (*n* = 1, also validated). PA levels were captured during the pre-pregnancy (*n* = 10) and early pregnancy (*n* = 9) stages.

Overall, PA was reported to be protective against GDM in 13 of 17 studies and the degree of protection generally increased with greater levels of PA. Eleven studies reported that PA before pregnancy was beneficially associated with reduced risk by 22–86% [36,37,39,40,44,49,51,56,61], with only two studies not reaching statistical significance [32,59]. The degree of potential protection depended on type and duration of PA. Similarly, ten studies that assessed early pregnancy PA levels also reported a reduction in GDM risk with higher PA, ranging between 11–52%, however two studies not reaching statistical significance [40,48]. When both pre-pregnancy and early pregnancy PA levels were taken into account, there was an even lower risk of GDM observed (RR = 0.31, 95% CI: 0.12–0.79) [40].

The most apparent associations between PA and GDM risk were observed in 13 studies reporting on Leisure Time PA (LTPA). Of these, ten reported a significant reduction in GDM risk [36,37,39,40,44,49,51,55,60,61]. Two studies that examined LTPA volumes, suggested ≥150 min/week [61] or ≥210 min/week [37] as being sufficient to reduce the risk of GDM. Higher leisure activity score [51] before pregnancy was associated with a 68% reduced risk of a high 1 h glucose challenge test. In terms of intensity-weighted PA volume (Metabolic Equivalent (MET) hours/week), the beneficial association of PA in the year before pregnancy were observed at ≥15 [44] or ≥21 MET hours/week [40]. Solomon et al. [32] and Van der Ploeg et al. [48] suggested a lower GDM risk with higher frequency and volume of vigorous PA, but this association did not reach statistical significance. In contrast, one study [52] reported that LTPA was associated with reduced rates of GDM only among women in the obese pre-pregnancy BMI category. Engaging in LTPA [39] before and during pregnancy was associated with a 46% GDM risk reduction.

The three studies that reported on total PA levels suggested that higher total PA volume [56,61] or score [59] was associated with lower risk of GDM [56,59,61], although one did not reach significance [59] and another was borderline statistically significant [56]. Two studies suggested that higher domestic (e.g., child and elderly caregiving, meal preparation, cleaning, shopping, gardening) PA levels were associated with lower risk of GDM [56,59], although one did not reach statistical significance [59]. Putman and colleagues observed that GDM risk was highest among women with the least average daily energy expenditure (≤2200 kcal) [53].

#### Meta-Analysis and Assessment of Bias

**Figure 3:**
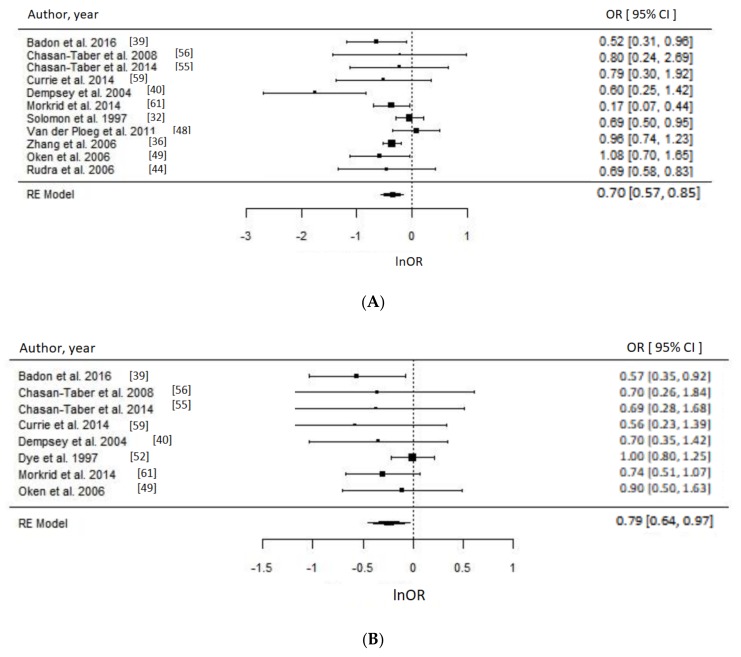
Metaanalysis of participation in any physical activity (PA) versus none and odds of gestational diabetes (GDM). Estimates are expressed as odds ratios (OR) with their corresponding 95% confidence intervals, however *x*-axis uses lnOR scale. (**A**) Engaging in any PA before pregnancy suggested 30% reduced odds of GDM (OR = 0.70, 95% CI = 0.57–0.85; *I*^2^ = 52% (Medium), *p*-value = 0.0006); (**B**) Engaging in any PA during early pregnancy suggested 21% lower odds of GDM (OR = 0.79, 95% CI = 0.64–0.97, *I*^2^ = 26% (low), *p*-value = 0.03).

**Figure 4:**
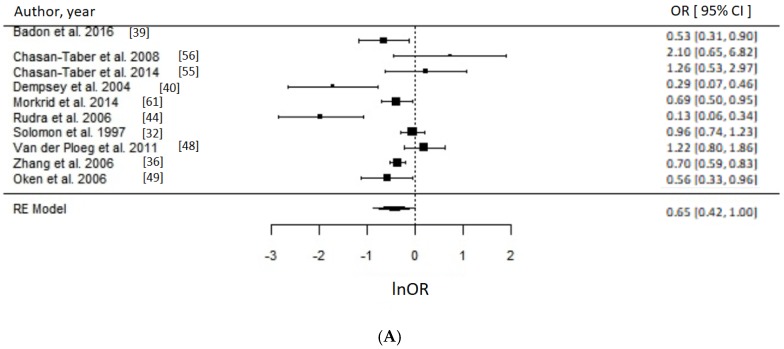

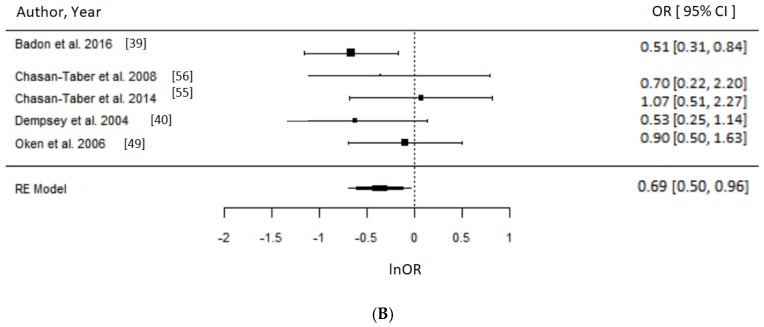
Metaanalysis of participation in high versus low level of leisure time physical activity (LTPA) and odds of gestational diabetes (GDM). Estimates are expressed as odds ratios (OR) with their corresponding 95% confidence intervals, however *x*-axis uses lnOR scale. (**A**) Engaging in any LTPA before pregnancy suggested possible reduced odds of GDM (OR = 0.65, 95% CI = 0.43–1.00; *I*^2^ = 90% (high), *p*-value = 0.05); (**B**) Engaging in any LTPA during early pregnancy suggests reduced odds of GDM (OR = 0.69, 95% CI = 0.50–0.96; *I*^2^ = 15% (low), *p*-value = 0.03).

Of 17 PA studies, 16 were suitable for meta-analyses [32,36,37,39,40,44,48,49,51,52,53,55,56,59,60,61] as they reported associations with sufficient statistical evidence. We were able to test a priori different indicators of PA and results were consistent. Engaging in any type of PA compared to none during the prepregnancy period was associated with approximately 30% reduced odds of GDM (OR = 0.70, 95% CI = 0.57–0.85; *I*^2^ = 52% (medium), *p*-value = 0.0006), whereas engaging in any PA early in pregnancy suggested reduced odds of GDM by 21% (OR = 0.79, 95% CI = 0.64–0.97, *I*^2^ = 26% (low), *p*-value = 0.03) as evident in Figure 3. Taking part in any LTPA compared to none either before (OR = 0.65, 95% CI = 0.43–1.00; *I*^2^ = 90% (high), *p*-value = 0.05) or during early pregnancy (OR = 0.69, 95% CI = 0.50–0.96; *I*^2^ = 15% (low), *p*-value = 0.03) suggested a beneficial association with GDM (Figure 4), with the prior not achieving statistical level of significance.

**Figure 5:**
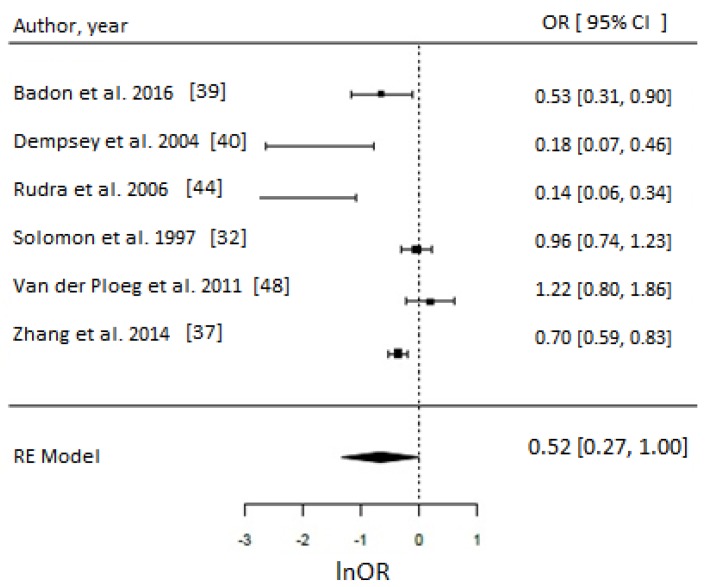
Metaanalysis of participation in high versus low level of leisure time physical activity (LTPA) before pregnancy in metabolic equivalents (MET.hr/week) and odds of gestational diabetes (GDM). Estimates are expressed as odds ratios (OR) with their corresponding 95% confidence intervals, however *x*-axis uses lnOR scale. Taking part in ~>15 MET.hr/week suggested 52% reduced odds of GDM (OR = 0.52, 95% CI = 0.27–1.00; *I*^2^ = 95%, *p*-value = 0.05). Due to insufficient number of studies reporting on LTPA in MET.hr/week in early pregnancy, a meta-analysis could not have been performed.

**Figure 6:**
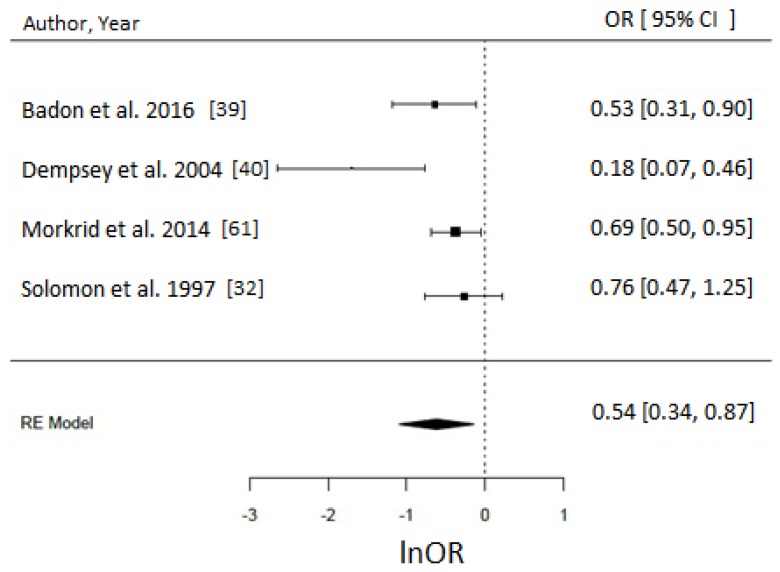
Meta-analysis of high versus low level of leisure time physical activity (LTPA) before pregnancy reported in hr/week and odds of gestational diabetes (GDM). Estimates are expressed as odds ratios (OR) with their corresponding 95% confidence intervals, however *x*-axis uses lnOR scale. Longer hours (>90 min/week) of LTPA/week reduced the odds of GDM by 46% (OR = 0.54, 95% CI = 0.34–0.87; *I*^2^ = 70% (medium), *p*-value = 0.01). Due to insufficient number of studies reporting on LTPA in hr/week in early pregnancy, a meta-analysis could not have been performed.

When comparing the studies reporting pre-pregnancy LTPA in MET.hr/week, our analysis suggested that >15 MET.hr/week was associated with 48% reduced odds of GDM (OR = 0.52, 95% CI = 0.27–1.00; *I*^2^ = 95%, *p*-value = 0.05) (Figure 5). Taking part in approximately >90 min/week in LTPA before pregnancy was associated with 46% reduced odds of GDM (OR = 0.54, 95% CI = 0.34–0.87; *I*^2^ = 70% (medium), *p*-value = 0.01) (Figure 6). It was not possible to perform a meta-analysis for the early pregnancy period for any LTPA indicator due to insufficient number of studies. For natural log OR values before back-transformation, refer to Table S4.

**Figure 7:**
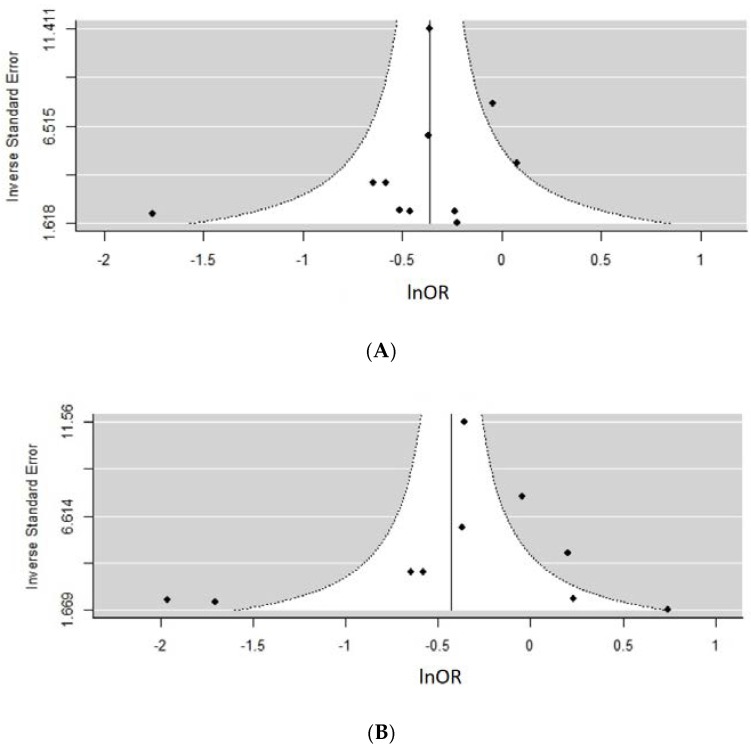
Assessing the risk of publication bias using funnel plots for different metaanalyses. (**A**) Any type pre-pregnancy physical activity (PA) versus none (*n* studies = 10, *z* = −1.52, *p* = 0.13). (**B**) Pre-pregnancy leisure time PA (LTPA), comparing high versus none regardless of units reported (*n* studies = 10, *z* = −0.65, *p* = 0.52) Due to insufficient number of studies reporting on early pregnancy period, a funnel plot could not have been performed for some meta-analyses.

Tests revealed a variable degree of heterogeneity ranging from 15–95%. Based on an almost symmetrical distribution of data points in funnel plots Figure 7A (*n* studies = 10, *z* = −1.52, *p* = 0.13) and Figure 7B (*n* studies = 10, *z* = −0.65, *p* = 0.52), there was no evidence of publication bias. Remaining funnel plots are presented in supplementary materials section as they contained <10 studies in their analyses. This includes Figure S1a (*n* studies = 9, *z* = −1.90, *p* = 0.06) with some symmetry present and Figure S1b (n studies = 6, z = −2.96, p = 0.003) and Figure S1c (*n* studies = 4, *z* = −2.34, *p* = 0.02) suggesting presence of asymmetry and potentially publication bias.

## 4. Discussion

The present systematic review identified 40 studies reporting on the relationship between diet or physical activity (PA) and subsequent risk of GDM. It is the first review to examine a range of specific dietary factors and indicators of PA, with a view to shedding light on the relative importance of these crucial lifestyle health behavior factors. We identified more observational studies than previous reviews [66,67], including those that additionally reported on PA levels, thereby providing a more comprehensive overview of lifestyle factors involved in the development of GDM. In addition, we visually depicted different types of confounding variables that were adjusted for in each individual study (Figure 2). While age, BMI and parity topped the list, we discovered that only one-in-three dietary studies adjusted for energy intake. This raises concerns about interpretation of data as many nutrients are associated with energy intake [68].

### 4.1. Diet and GDM Risk

Our present study investigated consumption of different types of beverages including fruit juice, SSB, coffee and tea intake in relation to GDM risk. While coffee consumption appeared to be protective against GDM in one study, SSB consumption resulted in a statistically significant positive association. There are now concerns over excessive SSB intake, particularly due to the reported association with obesity and risk of chronic diseases [69]. At the population level, there has been a decline in the overall SSB intake in several countries [69,70,71] over the same time frame, but there may be segments of the population such as young adults who continue to consume SSB in high amounts [72]. Added sugars from SSB are likely to be part of a poorer quality diet and lifestyle [73]. In one study [30], women with higher intake of SSB tended to have a diet lower in total dietary fiber, fruits and vegetables prompting the need to focus on a dietary pattern and quality in understanding any associations with GDM risk.

In particular, several studies from our literature search indicate that a MedDiet may be protective and possibly reduce the risk of GDM by 15–38%. The protective association may extend to reducing future risk of type 2 diabetes (by 19–23%), increasing chance of remission (by 49%) [74] and protection against cardiovascular diseases with extra virgin olive oil and plant-based dietary components in the diet [65]. There are however, drawbacks in defining what specifically constitutes a ‘traditional’ MedDiet due to variability among different regions of the Mediterranean. At present, a ‘traditional’ MedDiet is characterized by larger quantities of fruits, vegetables, legumes, nuts, unprocessed cereals and grains, extra virgin olive oil, moderate fish and wine and small amount of meat intake with low amounts of discretionary foods [65]. High red or processed meat consumption before pregnancy was associated with an increased risk of GDM. In fact, two meta-analyses conducted in a healthy adult population found that processed meat intake was associated with a greater risk of coronary heart disease (42%) and type 2 diabetes (19–32%) [75,76]. The proposed mechanism of coronary heart disease and type 2 diabetes onset include excess sodium and oxidative stress due to high levels of iron and advanced glycation end products [76] but warrants further discussion. Given the traditional MedDiet is low in meat consumption, this could be one of the reasons why the diet persistently provides health benefits across different age groups and stages of life.

The risk of GDM in an Australian population following a MedDiet was partly (32%) mediated by pre-pregnancy BMI [46]. While it cannot be denied that MedDiet provides multiple health benefits, the extent to which BMI explains the association with GDM comes as no surprise as obesity promotes insulin resistance [77]. In fact, a study by Janssen et al. [78] reported significant changes in insulin and leptin levels in the first trimester of women with a high BMI that was comparable to that of women with a normal BMI in the third trimester. This has enormous implications not only for maternal metabolism but also for fetal growth trajectory [78].

### 4.2. Physical Activity and GDM

The present meta-analysis suggested a protective association of PA (21–46%) from GDM when comparing any type of PA to none in either the pre-pregnancy or early pregnancy period. In a pooled data set from 6 studies, consisting of 661,137 men and women, Arem and colleagues [79] reported a similar protective association for all-cause mortality, which was steepest for comparisons between none (referent) and the equivalent of 150 min/week moderate-intensity LTPA (or 7.5 MET.hr/week). While current PA guidelines for pregnancy recommend 150 min/week [16,80] of moderate or 75 min/week vigorous intensity PA [81], the majority of adults still fail to meet the PA guidelines [82]

Our meta-analysis, however, suggests a potentially lower odds of GDM (46%) at >90 min/week. Similarly, O’Donovan and colleagues reported that taking part in 1 or 2 sessions/week in moderate-vigorous intensity PA resulted in CVD and all-cause mortality risk reduction regardless of an individual’s adherence to the current guidelines [83]. Whilst the potential benefits of structured LTPA are undisputed, volumes of PA below the recommended levels and even light intensity PA may have measurable health benefits [84].

We also observed that women who engaged in any type of PA compared to none in the year before pregnancy had potentially 10% lower odds of developing GDM than women who engaged in PA also compared to none during early pregnancy. The findings are further strengthened by presence of low-medium level of heterogeneity, no evidence of publication bias and data collected by predominantly validated questionnaires. On the other hand, Cordero and colleagues [85] suggested that engaging in structured PA 150–180 min/week during pregnancy could reduce the risk of GDM up to 90% when compared to standard care in their RCT. While it can be argued that study design may have an effect on the strength of the results, it cannot be denied that increasing PA appears to have an inverse association with risk of GDM. Since pregnancy is a temporary phase in a woman’s life, accompanied by many physiological and physical changes, preconception period should be perceived as a window of opportunity to adopt a healthier lifestyle. This could include incorporating more PA, achieving a healthy BMI and following a diet rich in plant-based food groups such as MedDiet to prevent undesirable pregnancy outcomes.

### 4.3. Strengths and Limitations

This study has strengths and limitations. Due to strict the selection criteria, a few larger population studies were excluded which may have limited the findings of this review. For example, the Coronary Artery Risk Development in Young Adults (CARDIA) study [86] (*n* = 1488) did not report on the relationship between diet or PA and risk of GDM, but rather differences in health behaviours between the pre-pregnancy period to several years after pregnancy. On the other hand, a study by Deierlein and colleagues [87] (*n* = 1437) did not report GDM as an outcome measure but used the term ‘hyperglycemia’ instead. Studies that were included in other systematic reviews [67], such as Saldana et al. [88] and He et al. [89], were excluded in our review due to late study recruitment and subsequently collection of dietary data that was not reflective of the early pregnancy period. Since all included studies are observational, they are susceptible to the effects of bias, confounding, potential measurement error, and under/over reporting of dietary intake. In the meta-analysis of PA, we were able to minimize these effects by using a random-effects model in statistical analysis (assuming heterogeneity) and in all the reviewed studies we conducted independent assessment of study quality, including whether validation of data collection methods had occurred (Table S2). What adds strength to the present study is that different indicators of LTPA were tested a priori with consistent findings. However, caution should be applied when interpreting sub-analysis of LTPA studies, particularly as there are <10 data sets available to be able to determine with confidence whether asymmetry is real or a coincidental occurrence [90].

## 5. Conclusions

Assuming that the associations we identified reflect causal relationships, our review suggests that a MedDiet and PA are promising interventions for the prevention of GDM. However, a greater degree of protection may occur when both lifestyle factors are incorporated before pregnancy and followed throughout pregnancy. Engaging in any PA even below the guidelines suggested a protective association with GDM risk. The finding in part raises the importance of individualized-patient care as the level of PA set in the current guidelines may be unachievable by some, however they could still potentially achieve similar health benefits at a lower PA threshold. There is an opportunity for future RCTs to further explore interventions with both PA and MedDiet pattern, especially in the pre-conception period to ensure best outcomes throughout pregnancy.

## Supplementary Materials

The following are available online at http://www.mdpi.com/2072-6643/10/6/698/s1, Table S1: Search Terms; Table S2: Modified quality assessment and risk of bias form obtained from the Evidence Analysis Manual: Steps in the academy evidence analysis process; Table S3: Characteristics of observational studies; Table S4: Natural odds ratio (lnOR) values before back-transformation correspond to Figure 3, Figure 4, Figure 5 and Figure 6. Figure S1: Assessing the risk of publication bias using funnel plots for different metaanalyses.
