# Supplementary material for: Associations of Diet and Physical Activity with Risk for Gestational Diabetes Mellitus: A Systematic Review and Meta-Analysis
Source: Nutrients. 2018 May 30;10(6):698. doi: 10.3390/nu10060698 (PMC6024719; doi:10.3390/nu10060698)
Supplement: Supplementary file 1 [file nutrients-10-00698-s001.pdf]

Supplementary Materials

Table S1. Search terms according to PICO formatting.

Aspect of PICO	No.	Term
Population	1	Pregnant
	2	Pregnancy/
	3	Pregnant women/
	4	Gestation$
	5	Maternal
	6	1 or 2 or 3 or 4 or 5
Intervention/ Observation (Food and Exercise)	7	Food intake/
	8	Food consumption
	9	Food habits
	10	Food analysis
	11	Dietary intake
	12	Macronutrient$.mp (dietary fat/ or dietary protein/ or dietary carbohydrate/)
	13	Calorie intake
	14	Energy intake
	15	Kilojoule intake
	16	Glycemic index
	17	Glycaemic index
	18	Glycemic load
	19	Glycaemic load
	20	Sugar$
	21	Potato
	22	White bread
	23	Soft drink$
	24	Sugar sweetened beverage$
	25	Soda
	26	Soda pop
	27	Carbonated drink
	28	Carbonated beverages
	29	Meat
	30	Meat products
	31	Meat intake
	32	Red meat
	33	Processed meat
	34	Dairy
	35	Dairy products
	36	Saturated fat
	37	Processed food$
	38	Pre-packaged food$
	39	Fast food

	40	Energy dense food$
	41	Convenience food
	42	Discretionary food$
	43	Discretionary snack$
	44	Snack$
	45	Physical Activity
	46	Exercise
	47	Movement
	48	Body movement
	49	Pedometer
	50	Active minutes
	51	Leisure time
	52	Resistance training
	53	Energy expenditure
	54	Energy metabolism
	55	7 or 8 or 9 or 10 or 11 or 12 or 13 or 14 or 15 or 16 or 17 or 18 or 19 or 20 or 21 or 22 or 23 or 24 or 25 or 26 or 27 or 28 or 29 or 30 or 31 or 32 or 33 or 34 or 35 or 36 or 37 or 38 or 39 or 40 or 41 or 42 or 43 or 44 or 45 or 46 or 47 or 48 or 49 or 50 or 51 or 52 or 53 or 54
**Outcome**	56	Gestational diabetes mellitus
	57	Gestational diabetes
	58	57 or 58
**Study type, Limitations**	59	Cohort
	60	Longitudinal
	61	Prospective
	62	60 or 61or 62
	63	Limit 63: English language, female, human and year limit (1985 – present)

Table S2 – Modified quality assessment & risk of bias form obtained from the Evidence Analysis Manual: Steps in the academy evidence analysis process.

Author, Year	Research question clearly stated	Participants representative of a GDM population	Response Rate	Attrition Rate	Exposure level described	Diet or PA assessment tools validated	Method of GDM diagnosis stated	Appropriate statistical analysis	Confounding factors adjusted	Discussion of findings, bias(es) & study limitations identified & discussed	Funding or sponsorship bias unlikely	Quality Rating
Adeney et al. 2007 [38]	Y	Y	*	Y	Y	X	Y	Y	Y	Y	Y	Neutral
Badon et al. 2016 [39]	Y	Y	NA	NA	Y	N	Y	Y	Not energy	Y	Y	Neutral
Bao et al. 2013 [24]	Y	Y	NA	NA	Y	Y	Y	Y	Y	Y	Y	Positive
Bao et al. 2014a [25]	Y	Y	NA	NA	Y	Y	Y	Y	Y	Y	Y	Positive
Bao et al. 2014b [26]	Y	Y	NA	NA	Y	Y	Y	Y	Y	Y	Y	Positive
Bao et al. 2016 [27]	Y	Y	NA	NA	Y	Y	Y	Y	Y	Y	Y	Positive
Baptiste-Robert et al. 2011 [51]	Y	Y	Y	NA	Y	Y	Y	Y	Not energy	Y	Y	Positive
Behboudi-Gandevani et al. 2013 [57]	Y	*	X	X	Y	Y	Y	Y	Not energy	Y	*	Neutral
Bowers et al. 2011 [28]	Y	Y	NA	NA	Y	Y	Y	Y	Y	Y	Y	Positive
Bowers et al. 2012 [29]	Y	Y	NA	NA	Y	Y	Y	Y	Y	Y	Y	Positive
Chasan-Taber et al. 2008 [56]	Y	Y	Y	Y	Y	Y	Y	Y	Not energy	Y	Y	Positive
Chasan-Taber et al. 2014 [55]	Y	Y	Y	Y	Y	Y	Y	Y	Not energy	Y	Y	Positive
Chen et al. 2009 [30]	Y	Y	NA	NA	Y	Y	Y	Y	Not energy	Y	Y	Neutral
Chen et al. 2012 [31]	Y	Y	NA	NA	Y	Y	Y	Y	Not energy	Y	Y	Neutral
Currie et al. 2014 [59]	Y	Y	Y	Y	Y	Y	Y	Y	Not Energy	Y	Y	Positive
Dempsey et al. 2004 [40]	Y	Y	Y	Y	Y	X	Y	Y	Not Energy	Y	Y	Positive
Dominguez et al. 2014 [62]	Y	Y	Y	X	Y	Y	Y	Y	Y	Y	*	Neutral
Dye et al. 1997 [52]	Y	Y	Y	Y	Y	X	Y	Y	Not Energy	Y	Y	Positive
Gresham et al. 2016 [45]	Y	Y	Y	*	Y	Y	Y	Y	Y	Y	Y	Positive
Harrison et al. 2012 [54]	Y	X	Y	Y	Y	Y	Y	Y	Not Energy	Y	Y	Positive
Hinkle et al. 2015 [58]	Y	Y	Y	Y	Y	*	*	Y	Not Energy	Y	*	Neutral
Iqbal et al. 2007 [60]	Y	Y	Y	Y	Y	Y	Y	Y	Not Energy	Y	Y	Positive
Karamanos et al. 2014 [63]	Y	Y	Y	X	Y	Y	Y	Y	Y	Y	Y	Positive
Morkrid et al. 2007 [61]	Y	Y	Y	Y	Y	Y	Y	Y	Not Energy	Y	Y	Positive
Oken et al. 2006 [49]	Y	Y	Y	Y	Y	Y	Y	Y	Not Energy	Y	Y	Positive
Osorio-Yanez et al. 2016 [41]	Y	Y	Y	Y	Y	Y	Y	Y	Y	Y	Y	Positive
Putnam et al. 2013 [53]	Y	Y	Y	X	Y	Y	Y	Y	Not Energy	Y	Y	Positive
Qiu et al. 2011a [42]	Y	Y	Y	Y	Y	Y	Y	Y	Y	Y	Y	Positive
Qiu et al. 2011b [43]	Y	Y	X	Y	Y	Y	Y	Y	Y	Y	Y	Positive
Radesky et al. 2008 [50]	Y	Y	Y	Y	Y	Y	Y	Y	Not Energy	Y	*	Neutral
Rudra et al. 2006 [44]	Y	Y	Y	X	Y	Y	Y	Y	Not Energy	Y	Y	Neutral

Author, Year	Research question clearly stated	Participants representative of a GDM population	Response Rate	Attrition Rate	Exposure level described	Diet or PA assessment tools validated	Method of GDM diagnosis stated	Appropriate statistical analysis	Confounding factors adjusted	Discussion of findings, bias(es) & study limitations identified & discussed	Funding or sponsorship bias unlikely	Quality Rating
Schoenacker et al. 2015 [47]	Y	Y	Y	Y	Y	Y	Y	Y	Y	Y	Y	Positive
Schoenacker et al. 2016 [46]	Y	Y	Y	Y	Y	Y	Y	Y	Y	Y	Y	Positive
Solomon et al. 1997 [32]	Y	Y	Y	X	Y	X	Y	Y	Not Energy	Y	Y	Neutral
Tobias et al. 2012 [33]	Y	Y	NA	NA	Y	Y	Y	Y	Y	Y	Y	Positive
Van der Ploeg et al. 2011 [48]	Y	Y	Y	X	Y	Y	Y	Y	Not Energy	Y	Y	Neutral
Zhang et al. 2006a [34]	Y	Y	NA	NA	Y	Y	Y	Y	Y	Y	Y	Positive
Zhang et al. 2006b [35]	Y	Y	NA	NA	Y	Y	Y	Y	Y	Y	Y	Positive
Zhang et al. 2006c [36]	Y	Y	NA	NA	Y	Y	Y	Y	Y	Y	Y	Positive
Zhang et al. 2014 [37]	Y	Y	NA	NA	Y	Y	Y	Y	Y	Y	Y	Positive

Key: Y = Yes; N = No; NA = Not Available; * = Unclear Abbreviations: GDM = Gestational Diabetes Mellitus; PA = Physical Activity

Table S3 – Characteristics of observational studies.

DIET & PHYSICAL ACTIVITY (PA)								
**Source**	**Aim & Study population**	**Selection Criteria**	**Diet Assessment Method**	**Physical Activity Assessment Method**	**Diagnostic Method for GDM**	**Statistical Analysis & Adjusted factors**	**Selected Main Findings (RR, OR etc.)**	**Quality Rating, Retention**
Baptiste-Robert et al. 2011 [51]	To determine pre-pregnancy PA & dietary intake in early pregnancy & its effect on glucose tolerance test.<br>n = 152<br>Age: 30.1 (SD = 5.2)<br>Country: United States<br>Study: Parity, Inflammation & DM	**Inclusion:** <14 wks gestation, no history of DM, consent to participate.	Validated Rapid Food Screener	Interview questionnaire (not validated)	50g, 1-hr GCT, Medical records	**Multiple logistic regressions**<br>**Adjustments:** race, age, parity, gestational weight gain & BMI.	68% less likely to have a 1-hr GCT response >140 mg/dL with a leisure score of ≥2.75 when compared to <2.75 [RR=0.32, 95% CI 0.12-0.86, P<0.05]. No association between dietary intake & response to 1-hr GCT response.	Positive, 64.4%
Zhang et al. 2014 [37]	To examine the effect of lifestyle characteristics on risk of GDM.<br>n women = 14 437<br>n pregnancies = 20 136<br>Age: 24-44<br>Country: United States<br>Study: NHS II	**Inclusion**: No history of GDM, T2DM, CVD & cancer.<br>**Exclusion**: Pregnancies after GDM.	Validated FFQ.	Validated physical activity questionnaire (not in a pregnant population).	Medical records	**Multivariable log binomial models with generalized estimating equations**<br>**Adjustments:** age, parity, family history of DM, history of infertility, race/ ethnicity, alcohol intake, questionnaire period & total EI.	Adhering to any 4 low risk lifestyle factors (AHEI-2010, PA, BMI, Smoking) before pregnancy, risk of GDM was lower by 83% when compared to those that did not adhere to any [RR=0.17, 95% CI 0.12-0.25]. Highest quintile of PA (≥210min/wk) vs lowest (<30min/wk) reduced the risk of GDM by 22% [RR=0.78, 95% CI 0.64-0.94].	Positive, NA%

DIET ONLY							
**Source**	**Aim & Study Population**	**Selection Criteria**	**Diet Assessment Method**	**Diagnostic method for GDM**	**Statistical Analysis & Adjusted factors**	**Selected Main Findings (RR, OR etc.)**	**Quality Rating, Retention**
Adeney et al. 2007 [38]	To examine the relationship between coffee consumption & the risk of GDM.<br>n = 1744<br>Age: 32.1 (0.1) yrs<br>Country: United States<br>Study: Omega	**Inclusion**: <16 wks gestation, knowledge of English language.<br>**Exclusion**: < 18 yrs, non-term pregnancy, did not plan to deliver at the research hospitals.	121-item semi-quantitative FFQ (not validated).	100g, 3-hr OGTT, Medical records	**Generalized linear model using a log-link function**<br>**Adjusted factors**: age, race, BMI, parity, smoking, alcohol use before pregnancy, smoking during pregnancy & chronic hypertension.	Moderate pre-pregnancy caffeinated coffee intake significantly reduced the risk of GDM by 52% when compared with non-consumers [RR=0.48, 95% CI 0.28-0.82].	Neutral, 87.2%
Bao et al. 2013 [24]	To examine the association between dietary protein & GDM.<br>n women = 15 294<br>n pregnancies = 21 457<br>Age: 25-44 years<br>Country: United States<br>Study: NHS II	**Inclusion**: singleton pregnancy, >6 months long, years 1991-2001.<br>**Exclusion:** Previous GDM, T2DM, cancer, CVD prior to pregnancy, FFQ not delivered or incomplete with unrealistic values.	Semi-quantitative FFQ (validated)	Medical records	**Multivariate logistic regression using generalized estimating equations**<br>**Adjustments:** age, parity, race/ethnicity, family history DM, smoking, alcohol intake, PA, total EI, intakes of saturated/monounsaturated/ trans/polyunsaturated fatty acids, dietary cholesterol, glycemic load, dietary fiber, mutual adjustment for animal protein & vegetable protein & BMI.	Animal protein intake significantly increased GDM risk by 49% [RR=1.49, 95% CI 1.03-2.17], whereas vegetable protein intake significantly reduced the risk of GDM by 31% [RR=0.69, 95% CI 0.50-0.97].	Positive, NA%
Bao et al. 2014a [26]	To examine the association between pre-pregnancy fried food consumption & risk of incident GDM.<br>n women = 15 027<br>n pregnancies = 21 079<br>Age: 25-44<br>Country: United States<br>Study: NHS II	**Inclusion**: No history of GDM, T2DM, cardiovascular disease & cancer.<br>**Exclusion:** no pre-pregnancy FFQ, an incomplete form or unrealistic EI (<600 or >3500kcal/day).	Semi-quantitative FFQ (validated)	Medical records	**Generalized estimating equations with log-binomials models**<br>**Adjustments**: age, parity, race/ ethnicity, family history of DM, smoking, PA, total EI, diet quality (AHEI-2010 score) & BMI.	Frequent fried food intake especially away from home, was associated with a greater risk of GDM when comparing frequency of ≥7/wk vs <1/wk [RR=2.18, 95% CI 1.53-3.09]. BMI adjustment resulted in attenuated but significant risk of GDM.	Positive, NA%

DIET ONLY							
**Source**	**Aim & Study Population**	**Selection Criteria**	**Diet Assessment Method**	**Diagnostic method for GDM**	**Statistical Analysis & Adjusted factors**	**Selected Main Findings (RR, OR etc.)**	**Quality Rating, Retention**
Bao et al. 2014b [25]	To examine the association of 3 pre-pregnancy low carbohydrate (CHO) diet patterns with risk of GDM.<br>n women = 15 265<br>n pregnancies = 21 411<br>Age: 25-44<br>Country: United States<br>Study: NHS II	**Inclusion**: No history of GDM, T2DM, CVD or cancer.<br>**Exclusion:** no pre-pregnancy FFQ or an incomplete form with unrealistic EI (<600 or >3500kcal/day).	Semi-quantitative FFQ (validated)	Medical records	**Log-binomials models with generalized estimating equation**<br>**Adjustments:** age, parity, race/ ethnicity, family history of DM, smoking, alcohol intake, PA, BMI & total EI.	Low CHO diet high in animal protein increases the risk of GDM by 36% [RR=1.36, 95% CI 1.13-1.64, P-trend= 0.003], however opposite is true for high vegetable protein & fat, reducing GDM by 16% [RR=0.84, 95% CI 0.69-1.03, P-trend=0.08]. Overall low CHO diet is associated with an increased risk of GDM [RR=1.27, 95% CI 1.06-1.51, P-trend=0.03].	Positive, NA%
Bao et al. 2016 [27]	To examine the association between pre-pregnancy potato consumption & risk of GDM.<br>n = 21 693<br>Age: 24-44<br>Country: United States<br>Study: NHS II	**Inclusion:** No history of GDM, T2DM, CVD or cancer.<br>**Exclusion:** no pre-pregnancy FFQ or an incomplete form with unrealistic EI (<600 or >3500kcal/day).	FFQ (validated)	Medical records	**Log-binomials models with generalized estimating equation.**<br>**Adjustments:** age, parity, race, family history of DM, smoking, PA, EI & AHEI-2010 score.	Consuming ≥5 servings/wk of potatoes compared to <1 serving/wk significantly increases the risk of GDM by 62% [RR=1.62, 95% CI 1.24-2.13, P<0.001].	Positive, NA
Behboudi-Gandevani et al. 2013 [57]	To investigate the association between maternal iron/zinc serum levels & women's nutritional intake in early pregnancy with GDM.<br>n = 1 033<br>Age: 27.57 (SD = 4.84)<br>Country: Iran	**Inclusion:** singleton pregnancy, 20-35 yrs, 14–20 wks gestation, attending prenatal clinics in specified hospitals.<br>**Exclusion**: disease of glucose metabolism (T1DM/T2DM), abortions, infections, chronic illness, or medical treatments.	Semi-quantitative FFQ (validated)	100g, 3-hr OGTT (2004 American Diabetes Association criteria)	**Mann–Whitney, chi-square & multiple logistic regression tests**<br>**Adjustments:** age, BMI, education, parity, passive smoking, history of GDM & family DM, serum zinc/iron & hemoglobin levels, & deficient zinc/iron intakes in early pregnancy.	Higher early pregnancy maternal serum iron levels increased risk of GDM [mean (SD) = 143.8 (48.7) versus 112.5 (83.5) μg/dL in GDM and non-GDM women respectively, P<0.0001]. No significant difference in zinc levels & iron/zinc nutritional intake between these groups [OR=1.006, 95% CI 1.002-1.009, P=0.001].	Neutral, NA%

DIET ONLY							
**Source**	**Aim & Study Population**	**Selection Criteria**	**Diet Assessment Method**	**Diagnostic method for GDM**	**Statistical Analysis & Adjusted factors**	**Selected Main Findings (RR, OR etc.)**	**Quality Rating, Retention**
Bowers et al. 2011 [28]	To determine if pre-pregnancy dietary & supplemental iron intakes are associated with risk of GDM.<br>n = 13 475<br>Age: 22-44<br>Country: United States<br>Study: NHS II	**Inclusion**: 22-44 yrs, singleton pregnancy, no history of GDM/T1DM/T2DM, CVD or cancer.<br>**Exclusion:** no pre-pregnancy FFQ, incomplete form, unrealistic EI (<600 or >3500kcal/day), peri-menopausal at baseline, missing information on age/iron intake.	133-item semi-quantitative FFQ (validated)	Medical records	**Pooled logistic regression, restricted cubic spline regressions**<br>**Adjustments**: Age, parity, BMI, PA, glycemic index, cereal fiber, polyunsaturated fatty acids, smoking status, alcohol, total calories, & family history of DM.	Dietary heme iron is positively associated with GDM risk when comparing highest vs lowest quintile [RR=1.58, 95% CI 1.21-2.08]. Every 0.5mg/day increase in heme iron intake increases risk of GDM by 22% [RR=1.22, 95% CI 1.10-1.36].	Positive, NA%
Bowers et al. 2012 [29]	To determine whether the total amount, type & source of pre-pregnancy dietary fats is related to risk of GDM.<br>n = 13 475<br>Age: 22-44<br>Country: United States<br>Study: NHS II	**Inclusion:** age 22-44 yrs, singleton pregnancy >6 months (1991-2001).<br>**Exclusion**: unrealistic total EI (<500 or 3500kcal/ day), DM, GDM, CVD, cancer, or missing information on age/iron intake or peri-menopausal at baseline.	133-item semi-quantitative FFQ (validated)	Medical records	**Pooled logistic regression**<br>**Adjustments**: age, parity, current smoking, BMI, PA, family history of DM, smoking, alcohol, race, & total EI, cereal fiber, dietary cholesterol, glycemic load & mutual adjustment for the specific fatty acids or source of fats.	Higher animal fat & cholesterol intakes increased GDM risk by 88% [RR=1.88, 95% CI 1.36-2.60, P=0.05] and 45% [RR=1.45, 95% CI 1.11-1.89, P=0.04] respectively, when comparing highest vs lowest quintile.	Positive, NA%
Chen et al. 2009 [30]	To examine the association between regular pre-gravid sugar sweetened beverage (SSB) consumption & the risk of GDM.<br>n = 13 475<br>Age: 24-44<br>Country: United States<br>Study: NHS II	**Exclusion**: Incomplete FFQ in 1991, >70 items left blank (FFQ), unrealistic total EI, multiple gestation, no PA data in 1991, history of DM, GDM, cancer or CVD.	133-item semi-quantitative FFQ (validated)	Medical records	**Cox proportional hazards models & multivariate adjustments**<br>**Adjustments**: age & parity.	Higher SSB significantly increased the risk of GDM by 23% when comparing ≥5 servings/wk vs <1/month [RR=1.23, 95% CI 1.05-1.45, P-value=0.005]. When SSB intake was treated as a continuous variable, each serving/day increment was associated with a 23% increase in GDM risk [RR=1.23, 95% CI 1.05-1.43, P-value=0.01].	Neutral, NA%

DIET ONLY							
**Source**	**Aim & Study Population**	**Selection Criteria**	**Diet Assessment Method**	**Diagnostic method for GDM**	**Statistical Analysis & Adjusted factors**	**Selected Main Findings (RR, OR etc.)**	**Quality Rating, Retention**
Chen et al. 2012 [31]	To examine the association of pre-pregnancy habitual consumption of fruits & fruit juices & GDM risk.<br>n = 13 475<br>Age: 22-44<br>Country: United States<br>Study: NHS II	**Inclusion**: women that did not have DM & major chronic diseases at baseline.	133-item semi-quantitative FFQ (validated)	Medical records	**Cox proportional hazards models & restricted cubic spline regressions**<br>**Adjustments:** age, parity, race, smoking, alcohol intake, PA, family history of DM, BMI, & dietary factors (cereal fiber, processed meat/red meat, SSB & fruit juice or apple).	Higher consumption of whole fruits is not associated with an increased GDM risk, when comparing highest vs lowest quintile [RR=0.93, 95% CI 0.76-1.16]. The association of fruit juices with GDM risk appears to be nonlinear, with lowest risk reported in women with moderate fruit juice consumption.	Neutral<br>NA%
Dominguez et al. 2014 [62]	To investigate the incidence of GDM according to the consumption of fast food in a cohort of university graduates.<br>n = 3 048<br>Country: Spain<br>Study: Seguimiento Universidad de Navarra (SUN)	**Inclusion:** Graduates from the University of Navarra & other Spanish universities, registered nurses & other health professionals from different Spanish provinces.<br>**Exclusion**: Extremely low/ high total EI, had previous GDM or DM.	Semi-quantitative FFQ (validated)	50g or 100g OGTT (2004 American Diabetes Association criteria)	**Non-conditional regression models**<br>**Adjustments**: age, total EI, smoking, PA, family history of DM, cardiovascular disease/ hypertension, parity, adherence to MedDiet pattern score, alcohol intake, fiber intake, and SSB intake and BMI.	Fast food consumption was significantly associated with an 86% higher risk of incident GDM when compared to the lowest category of fast food consumption [OR=1.86, 95% CI 1.13-3.06].	Neutral<br>97.2%
Gresham et al. 2016 [45]	To assess whether diet quality before or during pregnancy predicts adverse pregnancy & birth outcomes in Australian women.<br>n = 1 907<br>Age: 20.8 (SD 1.4)<br>Country: Australia<br>Study: Australian Longitudinal Study on Women's Health	**Exclusion**: not classified as pre-conception or pregnant when completing the FFQ, multiple birth, incomplete FFQ.	74-item FFQ (validated)	Self-report	**Multiple logistic regressions**<br>**Adjustments:** level of education, age, weight, area of residence, smoking status, parity, and level of exercise.	When comparing highest to lowest quintile, diet quality was not associated with GDM [OR=1.7, 95% CI 0.7-4.0].	Positive,<br>NA%

DIET ONLY							
**Source**	**Aim & Study Population**	**Selection Criteria**	**Diet Assessment Method**	**Diagnostic method for GDM**	**Statistical Analysis & Adjusted factors**	**Selected Main Findings (RR, OR etc.)**	**Quality Rating, Retention**
Hinkle et al. 2014 [58]	To examine the relation between first trimester coffee & tea intake & the risk of GDM.<br>n = 71 239<br>Age: 16-48 yrs<br>Country: Denmark<br>Study: Danish National Birth Cohort	**Inclusion**: first singleton pregnancy.<br>**Exclusion**: pre-existing DM, data of relevant covariates missing.	Interview	Self-report & medical records	**Chi-square statistics for bivariate analyses & modified Poisson regression**<br>**Adjustments**: age, parity, smoking status, cola intake, BMI, SES.	Suggested a protective, but non-significant association with increasing coffee [≥8 vs 0 cups/day RR=0.89, 95% CI 0.64-1.25] and tea intake [≥8 vs 0 cups/day RR=0.77, 95% CI 0.55-1.08].	Neutral, 82.4%
Karamanos et al. 2014 [63]	To investigate the association of MedDiet with the incidence of GDM in Mediterranean regions.<br>n = 1 003<br>Country: Algeria, France, Greece, Italy, Lebanon, Malta, Morocco, Serbia, Syria & Tunisia).	**Inclusion**: women with oral glucose tolerance test results, women with/without a history of GDM.<br>**Exclusion**: history of T1DM or T2DM.	Questionnaire (validated) & MedDiet Index.	75g, 1 & 2-hr OGTT (2010 International Association in Diabetes and Pregnancy Study Group criteria)	**Binary logistic regression**<br>**Adjustments**: age, BMI, family history of DM, gestational weight gain, EI.	GDM incidence was lower in subjects with better MedDiet adherence, 8.0% vs 12.3% [OR=0.62, 95% CI 0.40-0.95, P=0.030] by American Diabetic Association 2010 and 24.3% vs 32.8% [OR=0.66, 95% CI=0.50-0.87, P=0.004] according to International Association of Diabetes & Pregnancy Study Group 2012 criteria.	Positive 93.2%
Osorio-Yáñez et al. 2016 [41]	To examine the association between dietary Calcium intake and risk of GDM.<br>n = 3 414<br>Age: 32.8<br>Country: United States<br>Study: Omega	**Inclusion:** >18 yrs, <20 wks gestation, spoke & read English, delivered at specified hospitals.<br>**Exclusion:** history of DM/GDM, multi-gestation, pregnancy <20 wks, iron deficiency anaemia, incomplete FFQ, unrealistic levels of total EI (<500 kcal/day or >3500 kcal/ day).	121-item FFQ (validated)	100g, 3-hr OGTT (2004 American Diabetic Association criteria)	**Generalized linear models with log-link function, log Poisson regression model and robust standard errors.**<br>**Adjustments**: total energy, age, race/ethnicity, education, smoking status, BMI, prenatal vitamin use, PA, family history of DM, alcohol, coffee, SSB, red & processed meats, fatty fish, total fiber intake & dietary covariates (vitamin D & Mg).	Higher dietary Calcium intake compared to lower was inversely (though not statistically) associated with GDM risk [RR=0.57, 95% CI=0.27-1.21). Calcium intake ≥795 mg/day resulted in a 42% reduction in GDM risk when (<795 mg/day) [R=0.58, 95% CI 0.38-0.90, P-value= 0.02).	Positive, 74.2%

DIET ONLY							
**Source**	**Aim & Study Population**	**Selection Criteria**	**Diet Assessment Method**	**Diagnostic method for GDM**	**Statistical Analysis & Adjusted factors**	**Selected Main Findings (RR, OR etc.)**	**Quality Rating, Retention**
Qiu et al. 2011a [42]	To investigate the association of egg intake and dietary cholesterol & GDM risk in a cohort study.<br>n = 3 158<br>Age (mean): 32.7 yrs<br>Country: United States<br>Study: Omega	**Inclusion**: pre-natal care <20 wks, >18 yrs, spoke/read English, to deliver at either of 2 study hospitals.<br>**Exclusion**: DM, multi-gestation, incomplete or unrealistic dietary intake (<500 or >3500kcal/day).	121-item semi-quantitative FFQ (validated)	100g, 3-hr OGTT (2004 American Diabetic Association criteria)	**Multivariable models, generalized linear models using a log-link function**<br>**Adjustments**: EI, age, race/ethnicity, parity, PA, pre-pregnancy BMI, dietary fiber, vitamin C, intake red & processed meats, saturated fat intake.	Higher eggs and cholesterol intake during the pre-pregnancy and early pregnancy period were associated with a greater GDM risk [RR (≥10 eggs/week) = 2.52, 95% CI 1.11-5.72; RR (294 vs <151 mg/day cholesterol) = 2.35, 95% CI 1.35-4.09 respectively].	Positive, 79%
Qiu et al. 2011b [43]	To examine the associations of dietary heme & non-heme iron with the risk of GDM.<br>n = 3 158<br>Age: 32.7 yrs<br>Country: United States<br>Study: Omega	**Inclusion**: pre-natal care <20 wks, >18 yrs, spoke/read English, to deliver at either of 2 selected hospitals.<br>**Exclusion**: DM, multi-gestation, incomplete or excessive dietary intake (<500 or >3500kcal/day).	121-item semi-quantitative FFQ (validated)	100g, 3-hr OGTT (2004 American Diabetic Association criteria)	**Generalized linear models using a log-link function**<br>**Adjustments:** EI, age, race/ethnicity, parity, PA, pre-pregnancy BMI, dietary fiber, vitamin C.	Higher heme iron intake is associated with an increased GDM risk [RR=1.57, 95% CI 0.95–2.61] when comparing highest to quartile. Women who reported very high heme iron intake (≥1.52 mg/ day) had a 2.26-fold increased risk (95% CI 1.09–4.69) of GDM compared with women reporting lower levels.	Positive, 79%
Radesky et al. 2008 [48]	To report results from an analysis of diet quality & risk of abnormal glucose tolerance among a cohort of women.<br>n = 1 733<br>Age: 32.2 (4.9) yrs<br>Country: United States<br>Study: Project Viva	**Inclusion**: <20 wks, singleton pregnancy, complete study forms in English.<br>**Exclusion**: missing or incomplete oral glucose tolerance test & diet, history of T2DM or T2DM, or polycystic ovarian syndrome.	Self-administered Semi-quantitative FFQ (validated)	100g, 3-hr OGTT (2004 American Diabetes Association criteria)	**Multinomial regression**<br>**Adjustments**: age, pre-pregnancy BMI, race/ ethnicity, family history of DM, history of GDM.	Alpha-linolenic acid was associated with increased risk for GDM [OR=1.29, 95% CI 1.04-1.60) for each 300 mg/day after adjustment for confounders & other fats. Overall women with GDM had higher average n-3 fatty acid intake, lower n-6/n-3 ratio, and slightly higher polyunsaturated fat intake than normo-glycaemic women.	Neutral, 81.4%

DIET ONLY							
**Source**	**Aim & Study Population**	**Selection Criteria**	**Diet Assessment Method**	**Diagnostic method for GDM**	**Statistical Analysis & Adjusted factors**	**Selected Main Findings (RR, OR etc.)**	**Quality Rating, Retention**
Schoenacker et al. 2015 [47]	To examine the associations between pre-pregnancy dietary patterns & risk of GDM.<br>n = 3 853<br>n pregnancies = 6626<br>Age: 28 (1.4) yrs<br>Country: Australia<br>Study: Australian Longitudinal Study on Women's Health	**Inclusion**: Australian women without pre-existing DM.<br>**Exclusion**: T2DM or T2DM, pregnant with their first child in 2003, did not report a live birth at consecutive surveys in 2006/2009/2012, missing data, had GDM, unrealistic EI (<2093 or >14654kJ/d).	Questionnaire (validated)	75g, 1-hr OGTT;<br>Self-report (1998 Australasian Diabetes in Pregnancy Society criteria)	**Generalized estimating equation**, **Log-binomial models or Log-Poisson**<br>**Adjustments**: age, EI, parity, hypertensive disorders of pregnancy, highest education, smoking status, PA, BMI, polycystic ovarian syndrome.	No association between fruit & low-fat dairy or cooked vegetables with GDM risk. Mediterranean-style diet associated with 15% lower GDM risk [RR= 0.85, 95% CI 0.76-0.98]. Each SD increase in score of the meats, snacks & sweets pattern was associated with 41% higher GDM risk [RR= 0.59, 95% CI 1.03-1.91]. This association was no longer statistically significant after additional adjustment including BMI [RR=1.35, 95% CI 0.98-1.81].	Positive, 42.4%
Schoenacker et al. 2016 [46]	To determine how much pre-pregnancy BMI mediates the association between a pre-pregnancy MedDiet & development of GDM.<br>n = 3 378<br>Country: Australia<br>Study: Australian Longitudinal Study on Women's Health	**Inclusion:** not pregnant at baseline and who reported ≥1 live birth during the 9-y follow-up.<br>**Exclusion:** women in rural or remote areas.	FFQ (validated)	Self-report (1998 Australasian Diabetes in Pregnancy Society criteria)	**Linear or logistic regression**<br>**Adjustments**: education, parity, polycystic ovarian syndrome, EI and PA.	BMI contributes 32% to the total effects and relationship between pre-pregnancy MedDiet and odds of GDM [OR=1.35, 95% CI 1.02-1.60].	Positive, 84.5%

DIET ONLY							
**Source**	**Aim & Study Population**	**Selection Criteria**	**Diet Assessment Method**	**Diagnostic method for GDM**	**Statistical Analysis & Adjusted factors**	**Selected Main Findings (RR, OR etc.)**	**Quality Rating, Retention**
Tobias et al. 2012 [33]	To assess usual pre-pregnancy adherence to well-known dietary patterns & GDM risk.<br>n = 15 254<br>Age: 24-44<br>Country: United States<br>Study: NHS II	**Inclusion**: singleton pregnancy, no GDM history, no history of DM/cancer/ CVD event.<br>**Exclusion**: pregnancies after GDM, pre-pregnancy FFQ, left >70 FFQ items blank, or reported unrealistic total EI (<500 or 3500kcal/ day).	Semi-quantitative FFQ (validated)	Medical records	**Multi-variable marginal logistic using Generalized estimating equation**<br>**Adjustments**: age, EI, race/ethnicity, PA, BMI, family history of DM, gravidity, smoking status.	Comparing high to low dietary adherence, the risk of GDM was 24% lower with the alternate MedDiet score [RR=0.76, 95% CI 0.60, 0.95, P-value = 0.004], 34% lower with the Dietary Approaches to Stop Hypertension (DASH) score [RR=0.66, 95% CI 0.53, 0.82, P-trend=0.0005], & 46% lower with the AHEI score [RR=0.54, 95% CI 0.43, 0.68, P-trend=<0.0001].	Positive, NA%
Zhang et al. 2006a [34]	To examine whether pre-gravid dietary fiber consumption from cereal, fruit, & vegetable sources & dietary glycemic load was related to GDM.<br>n = 13 110<br>Age: 24-44<br>Country: United States<br>Study: NHS II	**Inclusion**: pregnant women.<br>**Exclusion**: did not complete FFQ in 1991, incomplete FFQ, dietary intake was unrealistic total EI (500 kcal/day or 3,500 kcal/day), multiple gestation or history DM/cancer/CVD or GDM.	133-item Semi-quantitative FFQ (validated)	Medical records	**Cox proportional hazards analysis**<br>**Adjustments**: parity, age, BMI, smoking status, race/ ethnicity, PA, family history of DM & dietary variables (total fat expressed as % energy), cereal fiber, fruit & vegetable fiber, alcohol consumption, EI & glycaemic load.	Dietary total fiber & cereal & fruit fiber were strongly inversely associated with GDM risk. Each 10g/day increment in total fiber intake was associated with 26% (RR=0.74, 95% CI 0.51-0.91) reduction in risk. Each 5g/day increment in cereal or fruit fiber was associated with a 23% (9 –36) or 26% (5– 42) reduction respectively.	Positive, NA%
Zhang et al. 2006b [35]	To examine whether dietary patterns are related to risk of GDM.<br>n = 13 110<br>Age: 24-44<br>Country: United States<br>Study: NHS II	**Inclusion**: pregnant women<br>**Exclusion**: did not complete FFQ in 1991, > 9 items blank in FFQ, unrealistic total EI (500 kcal/day or 3,500 kcal/day), multiple gestation or history of DM/cancer/CVD or GDM.	133-item semi-quantitative FFQ (validated)	Medical records	**Cox proportional hazards analysis**<br>**Adjustments**: parity, age, BMI, smoking status, race/ ethnicity, PA, family history of diabetes & dietary variables including total fat (% energy), cereal fiber, alcohol intake, total EI & glycaemic load.	Comparing the *highest* with the *lowest* quintile of the Western pattern scores, RR=1.63 (95% CI 1.20–2.21, P=0.001) & conversely comparing the lowest with the highest quintile of the prudent pattern scores, RR=1.39 (95% CI 1.08–1.80, P=0.018).	Positive, NA%

Source	Aim & Study Population	Selection Criteria	Physical Activity Assessment Method	Diagnostic method for GDM	Statistical Analysis & Adjusted factors	Selected Main Findings (RR, OR etc.)	Quality Rating, Retention
**PHYSICAL ACTIVITY ONLY**							
Badon et al. 2016 [39]	To investigate the associations of Leisure Time Physical Activity (PA) before and during pregnancy with GDM risk.<br>n = 3 449<br>Age: 32.6 (SD 4.4)<br>Country: United States<br>Study: Omega	**Inclusion**: >18 yrs, speak & read in English language, prenatal care <20 wks gestation, deliver at allocated hospitals.<br>**Exclusion**: Pre-pregnancy or early pregnancy PA of ≥35 metabolic equivalents (MET-hrs/week), missing data on PA, had prior T1/T2DM.	Questionnaire (Invalidated)	100-g, 3-hr OGTT (1997 American Diabetic Association criteria)	**Multivariable Poisson regression**<br>**Adjustments**: age, race, education, marital status, nulliparity, pre-pregnancy BMI category, gestational weight gain, smoking during pregnancy, alcohol use during pregnancy & year of study enrollment.	Leisure time PA during both pre-pregnancy and early pregnancy was associated with a 46% reduced risk of GDM [RR=0.54, 95% CI 0.32-0.89] when compared with inactivity.	Neutral, NA
Chasan-Taber et al. 2008 [56]	To determine whether PA during pregnancy reduces the risk of GDM in Hispanic women.<br>n = 1006, (710 for mid-pregnancy data)<br>Age: 16-40 yrs<br>Country: United States	**Inclusion:** age 16-40 yrs, <24 wks gestation.<br>**Exclusion**: Non-Hispanic, T2DM, hypertension, heart disease, chronic renal disease, medications that influence glucose tolerance, multi-gestation & previous participation in the study.	Kaiser PA Survey & Pregnancy PA Questionnaire (validated in a pregnant population)	100g, 3 hr OGTT (2004 American Diabetic Association criteria), medical records	**Logistic regression**<br>**Adjustments**: age & BMI.	Higher levels of household/ caregiving activity in early (OR=0.2, 95% CI 0.1-0.8, P-trend=0.03) & mid-pregnancy (OR=0.2, 95% CI 0.1-0.8, P-trend=0.004) were associated with a reduced risk of GDM. Higher level of total PA was also associated with reduced odds of GDM (OR=0.4, 95% CI 0.1-1.2, P-trend=0.06).	Positive, 81.7%
Chasan-Taber et al. 2014 [55]	To examine the relationship between PA during pre, early & mid pregnancy & risk of abnormal glucose tolerance & GDM.<br>n = 1241<br>Age: 16-40 yrs<br>Country: United States<br>Study: Proyecto Buena Salud	**Inclusion**: born in the Caribbean Islands or had a parent or ≥2 grand-parents born in the Caribbean Islands.<br>**Exclusion**: history of DM/hypertension/heart or renal disease, <16 or >40 yrs old, multi- gestation or medications that influence glucose tolerance.	Pregnancy PA Questionnaire (validated in pregnant women)	100g, 3-hr OGTT (2004 American Diabetic Association criteria); medical records	**Logistic regression**<br>**Adjustments**: age, BMI, gestational weight gain, education level, generation in the United States.	Women in the top quartile of moderate intensity PA in early pregnancy had a 52% decreased risk of abnormal glucose result when compared to the lowest quartile [OR=0.48, 95% CI 0.27-0.88, P-trend=0.03]	Positive, 76.3%

PHYSICAL ACTIVITY ONLY							
**Source**	**Aim & Study Population**	**Selection Criteria**	**Physical Activity Assessment Method**	**Diagnostic method for GDM**	**Statistical Analysis & Adjusted factors**	**Selected Main Findings (RR, OR etc.)**	**Quality Rating, Retention**
Currie et al. 2014 [59]	To examine if physical activity in the year pre- pregnancy & in the first half of pregnancy is associated with maternal & neonatal outcomes.<br>n = 1 749<br>Age: 31 (mean)<br>Country: Canada	**Exclusion**: >20 wks gestation, pre-existing DM, early pregnancy loss or pregnancy termination, any missing information, contraindications to PA present before 20 wks gestation.	Kaiser PA Survey (validated in pregnant women)	50g, 1-hr GCT or 100g 1 & 2-hr OGTT, Medical records	**Logistic regression**<br>**Adjustments**: age, pre-pregnancy BMI, education, parity, & history of GDM.	Relative to the lowest tertile of pre-pregnancy household PA, women in the middle & the highest tertiles were at decreased risk of GDM [OR=0.29, 95% CI 0.12 – 0.74 & OR=0.33, 95% CI 0.12 - 0.88] respectively, albeit statistically insignificant.	Positive, 79.5%
Dempsey et al. 2004 [40]	To examine the relationship between recreational PA before & during pregnancy & risk of GDM.<br>n = 909<br>Country: United States<br>Study: Omega	**Inclusion**: <16 wks gestation<br>**Exclusion**: <18 yrs, did not speak/read English, did not carry to term, if they did not plan to deliver at the selected hospitals.	Questionnaire (Invalidated)	100-g, 3-hr OGTT (1997 American Diabetes Association criteria), Medical records	**Generalized linear models using a log-link function**<br>**Adjustments:** maternal age, race, parity, & pre-pregnancy BMI.	Compared with those who were inactive, women who participated in any recreational PA in the pre-pregnancy period, had a 56 % GDM risk reduction (RR=0.44, 95% CI 0.21 - 0.91). Women who engaged in PA before & during pregnancy had a 69% GDM reduced risk (RR=0.31, 95% CI 0.12, 0.79).	Positive, 90.9%
Dye et al. 1997 [52]	To determine whether exercise has a preventive role in the development of GDM in women living in central New York State on a population-based birth registry.<br>n = 12 799<br>Country: United States	**Inclusion**: women that delivered a livebirth within the New York State between 1/10/1995-31/07/1996.<br>**Exclusion**: conditions that affect exercise (e.g. heart disease, multi-gestation, incompetent cervix, previous preterm delivery & low birth weight infant & chronic hypertension).	Personal interview	Medical records	**Chi-square statistics, Logistic regression**<br>**Adjustments**: age, race, parity, pre-pregnancy BMI, gestational weight gain & insurance coverage.	When stratified by pre-pregnancy BMI category, exercise was associated with reduced rates of GDM only among women with a BMI >33 [OR=1.9, 95% CI 1.2-3.1].	Positive, 89.1%

Source	Aim & Study Population	Selection Criteria	Physical Activity Assessment Method	Diagnostic method for GDM	Statistical Analysis & Adjusted factors	Selected Main Findings (RR, OR etc.)	Quality Rating, Retention
**PHYSICAL ACTIVITY ONLY**							
Iqbal et al. 2007 [60]	To identify lifestyle predictors of GDM in South Asian women.<br>n = 611<br>Age: 29.4 (4.7)<br>Country: Canada	**Inclusion:** women of South Asian origin, ≤18 wks of gestation & did not have known diabetes.<br>**Exclusion**: missing data, terminating a pregnancy, refusing oral glucose tolerance test.	Interviewer administered Monitoring Trends & Determinants of Cardiovascular Disease (Monica) Optional Study of PA, (Validated)	100g, 3-hr OGTT (2004 American Diabetic Association criteria)	**Logistic regression**<br>**Adjustments:** age, family history of DM, education, height, parity BMI, PA level (kcal/day) & rate of weight gain/wk.	Increase in PA (100 kcal), decreased the risk of GDM by 11% [OR=0.89, 95% CI 0.79-0.99].	Positive, 81.6%
Morkrid et al. 2007 [61]	To assess the association between objectively recorded PA in early gestation & GDM identified at multiethnic cohort.<br>n = 759<br>Age: 29.9 (4.4)<br>Country: Norway<br>Study: Stork Groruddalen Study	**Inclusion**: lived in one of the selected districts, to give birth in one of the 2 selected hospitals, <20 wks gestation, could speak one of the 9 listed languages & to provide written consent.<br>**Exclusion**: known diabetes or other diseases requiring frequent hospital visits.	Questionnaire (validated)	75g, 2-hr OGTT (amended 2010 International Association of Diabetes & Pregnancy Study Group criteria)	**Logistic regression**<br>**Adjustments:** ethnic origin, wks gestation, age, parity, & pre-pregnancy BMI.	Significant associations between the following 3 components GDM risk: objectively recorded steps/day in early gestation [OR=0.79, 95% CI 0.65 –0.97], self-reported regular PA before pregnancy [OR=0.66, 95% CI 0.46-0.94] & self-reported aerobic PA ≥ 150 min/wk 3 months before pregnancy [OR=0.69, 95% CI 0.49-0.97].	Positive, 92.2%

PHYSICAL ACTIVITY ONLY							
**Source**	**Aim & Study Population**	**Selection Criteria**	**Physical Activity Assessment Method**	**Diagnostic method for GDM**	**Statistical Analysis & Adjusted factors**	**Selected Main Findings (RR, OR etc.)**	**Quality Rating, Retention**
Oken et al. 2006 [49]	To examine the associations of PA & television viewing before & during pregnancy, with risk for GDM & abnormal glucose tolerance.<br>n = 1 805<br>Age: 32.1 (5.0)<br>Country: United States<br>Study: Project Viva	**Exclusion:** history of T1DM or type 2 diabetes no measurement of blood glucose levels during pregnancy, no data on PA or TV viewing, no records of pre-pregnancy BMI.	Questionnaire; modified from the leisure time activity section of the PA Scale for the Elderly (validated on an elderly population).	100g, 3-hr OGTT (2004 American Diabetic Association criteria)	**Logistic regression Adjustments:** age, race/ethnicity, pre-pregnancy BMI, history of GDM in a previous pregnancy, & mother's history of DM.	Vigorous activity during the year before pregnancy reduced the risk of GDM by 44% [OR=0.56, 95% CI 0.33-0.95]. Vigorous activity before pregnancy & light-to-moderate or vigorous activity during pregnancy appeared to reduce the risk of GDM [OR=0.49, 95% CI 0.24-1.01].	Positive, 84.8%
Putnam et al. 2013 [53]	To determine association between daily physical activity & pregnancy & neonatal outcomes in stay at home military wives.<br>n = 190<br>Age: 28.3 (5.5)<br>Country: United States	**Inclusion:** unemployed, married to an active-duty or reserve service member, aims to complete prenatal care & delivery within the specified medical facility.<br>**Exclusion**: preexisting hypertension/ DM or thrombophilia, multiple gestation, or history of preterm delivery.	Validated questionnaire describing their domestic PA on a typical day during the previous 4 weeks (1st trimester).	100g, 3-hr OGTT, no further information	**Logistic regression Adjustments**: maternal BMI at first visit & delivery, number of children at home, gravidity, & parity.	Highest incidence rate of GDM occurred in the group with the least average daily energy expenditure (P=0.025).	Positive, NA%

PHYSICAL ACTIVITY ONLY							
**Source**	**Aim & Study Population**	**Selection Criteria**	**Physical Activity Assessment Method**	**Diagnostic method for GDM**	**Statistical Analysis & Adjusted factors**	**Selected Main Findings (RR, OR etc.)**	**Quality Rating, Retention**
Rudra et al. 2006 [44]	To examine the relation between perceived exertion & GDM within sub-groups of women categorize by energy expenditure.<br>n = 897<br>Country: United States<br>Study: Omega	**Inclusion:** women who initiated prenatal care before 16 wks gestation.<br>**Exclusion**: <18 yrs, did not speak/read English, did not plan to carry the pregnancy to term, or did not plan to deliver at either of the specified hospitals.	Stanford 7-Day PA Recall & the Minnesot Leisure-Time PA Questionnaire, (validated among men & non-pregnant women).	Medical records	**Logistic regression models**<br>**Adjustments**: age, race/ethnicity pre-pregnancy hypertension, nulliparity, & pre-pregnancy BMI.	Women reporting strenuous & very strenuous maximal exertion had 37% [OR=0.63, 95% CI 0.31-1.29] & 43% [OR=0.57, 95% CI 0.24-1.37] lower risk of GDM respectively, when compared with negligible-moderate exertion. Women reporting ≥15.0 MET-hours/week experienced 86% GDM risk reduction when compared to inactive women [OR=0.14, 95% CI 0.05-0.38].	Neutral, 89.7%
Solomon et al. 1997 [32]	To assess whether recognized determinants of NIDDM may also be markers for increased risk of GDM.<br>n = 14 613<br>Age: 25-42 yrs<br>Country: United States<br>Study: NHS II	**Inclusion**: no history of GDM or diabetes, singleton pregnancy between 1990 & 1994, pregnancy lasting >6 months.<br>**Exclusion**: multiple pregnancy.	PA (1989) - assessed as average MET expenditures. In 1991 - women were questioned about the number of times /wk they engaged in PA to perspire heavily.	Medical records	**Logistic regression**<br>**Adjustments**: age, BMI & parity.	No association between total MET score in 1989 & subsequent GDM risk. GDM risk appeared slightly lower with frequent participation in vigorous PA, albeit statistically insignificant [RR(≥4/week) = 0.78, 95% CI 0.47-1.29].	Neutral, NA%

PHYSICAL ACTIVITY ONLY							
**Source**	**Aim & Study Population**	**Selection Criteria**	**Physical Activity Assessment Method**	**Diagnostic method for GDM**	**Statistical Analysis & Adjusted factors**	**Selected Main Findings (RR, OR etc.)**	**Quality Rating, Retention**
Van der Ploeg et al. 2011 [48]	To examine the relationships between PA, sedentary behavior & the development of GDM<br>n = 3 529<br>Age: 24-34 yrs<br>Country: Australia<br>Study: Australian Longitudinal Study on Women's Health	**Inclusion:** Women in Australia.<br>**Exclusion:** T1DM, type 2 diabetes were pregnant at the second survey, were pregnant with their first child at the third survey or did not have a live-birth between survey 2 & 3.	Australian Longitudinal Study on Women's Health modification of the 7-day recall Active Australia questionnaire. Non-validated	75 g, 2-hr OGTT, self-reported (1998 Australasian Diabetes in Pregnancy Society criteria).	**Generalized estimating equations**<br>**Adjustments**: EI, overweight & obesity, age, BMI, parity, age at birth of first child, country of birth, & education.	Neither total PA nor sedentary behavior were associated with the risk of GDM. Analyses for self-reported vigorous PA showed no significant relationships with the development of GDM, with OR=1.23 [95% CI 0.83-1.81] & OR=0.95 [95% CI 0.62-1.46] for 1–90 min/wk & >90 min/wk, respectively.	Neutral,<br>S2: 82.0%<br>S3: 75.3%
Zhang et al. 2006c [36]	To assess whether the amount, type, & intensity of pre-gravid PA & sedentary behaviors are associated with GDM risk.<br>n = 21 765<br>Age: 24-44 yrs<br>Country: United States<br>Study: NHS II	**Inclusion**: singleton pregnancy lasting 6 months or longer.<br>**Exclusion**: history of GDM/diabetes/cancer or cardiovascular disease, were pregnant in 1989 questionnaire, no PA data, multiple gestation.	Questionnaire (validated)	Medical records	**Cox proportional hazards analysis**<br>**Adjustments**: parity, nulliparous women, age, smoking status, race or ethnicity, family history of diabetes & dietary variables (total fat, % energy, cereal fiber, alcohol, GI, total EI) & BMI.	Highest quintile of vigorous PA significantly reduced the risk of GDM by 23%, when compared to the lowest quintile [RR=0.77, 95% CI 0.69-0.94, P-trend=0.002].	Positive 69.8%

Abbreviations: AHEI-2010 – Alternative Healthy Eating Index – 2010, BMI – Body mass index, CHO – Carbohydrates, CIs – confidence intervals, CVD – Cardiovascular Disease, DM – Diabetes Mellitus, EI – Energy Intake, FFQ – food frequency questionnaire, GCT – Glucose Challenge Test, GDM – gestational diabetes mellitus, GI – Glycemic index MedDiet – Mediterranean Diet, MET - metabolic equivalent, NA – Not Available, NHS I/II – Nurse's Health Study I or II, NIDDM – non-insulin dependent diabetes mellitus, OGTT – Oral Glucose Tolerance Test, OR – odds ratio, PA – physical activity, RCT – randomized controlled trial, RR – relative risk, SD – Standard Deviation, SE – Standard Error, SES – socioeconomic status, SSB – Sugar sweetened beverage, T1/T2DM – Type 1 or Type 2 Diabetes Mellitus,

Table S4. Natural odds ratio (lnOR) values before back-transformation correspond to Figures 3A, 3B, 4A, 4B, 5 and 6.

	Study	lnOR	95 % Confidence Intervals	
			Lower	Upper
Figure 3A	Badon et al. 2016 [39]	-0.64	-1.18	-0.11
	Chasan-Taber et al. 2008 [56]	-0.22	-1.43	0.99
	Chasan-Taber et al. 2014 [55]	-0.24	-1.12	0.65
	Currie et al. 2014 [59]	-0.51	-1.37	0.35
	Dempsey et al. 2004 [40]	-1.76	-2.69	-0.83
	Morkrid et al. 2014 [61]	-0.37	-0.70	-0.05
	Solomon et al. 1997 [32]	-0.04	-0.30	0.21
	Van der Ploeg et al. 2011 [48]	0.08	-0.35	0.50
	Zhang et al. 2006 [36]	-0.36	-0.54	-0.19
	Oken et al. 2006 [49]	-0.58	-1.12	-0.04
	Rudra et al. 2006 [44]	-0.46	-1.34	0.42
	OVERALL	-0.36	-0.57	-0.16
Figure 3B	Badon et al. 2016 [39]	-0.56	-1.04	-0.08
	Chasan-Taber et al. 2008 [56]	-0.36	-1.33	0.61
	Chasan-Taber et al. 2014 [55]	-0.37	-1.26	0.52
	Currie et al. 2014 [59]	-0.58	-1.49	0.33
	Dempsey et al. 2004 [40]	-0.35	-1.04	0.35
	Dye et al. 1997 [52]	0.00	-0.22	0.22
	Morkrid et al. 2014 [61]	-0.30	-0.67	0.07
	Oken et al. 2006 [49]	-0.11	-0.70	0.49
	OVERALL	-0.24	-0.45	-0.03
Figure 4A	Badon et al. 2016 [39]	-0.64	-1.18	-0.11
	Chasan-Taber et al. 2008 [56]	0.74	-0.43	1.92
	Chasan-Taber et al. 2014 [55]	0.23	-0.63	1.09
	Rudra et al. 2006 [44]	-1.71	-2.64	-0.77
	Dempsey et al. 2004 [40]	-0.37	-0.70	-0.05
	Morkrid et al. 2014 [61]	-1.97	-2.86	-1.07
	Solomon et al. 1997 [32]	-0.04	-0.30	0.21
	Van der Ploeg et al. 2011 [48]	0.20	-0.22	0.62
	Zhang et al. 2006 [36]	-0.36	-0.53	-0.19
	Oken et al. 2006 [49]	-0.58	-1.12	-0.04
	OVERALL	-0.43	-0.86	0.00

Figure 4B	Badon et al. 2016 [39]	-0.67	-1.16	-0.18
	Chasan-Taber et al. 2008 [56]	-0.36	-1.50	0.79
	Chasan-Taber et al. 2014 [55]	0.07	-0.68	0.82
	Dempsey et al. 2004 [40]	-0.63	-1.39	0.13
	Oken et al. 2006 [49]	-0.11	-0.70	0.49
	OVERALL	-0.37	-0.70	-0.04
Figure 5	Badon et al. 2014 [39]	-0.64	-1.18	-0.11
	Dempsey et al. 2004 [40]	-1.71	-2.64	-0.77
	Rudra et al. 2006 [44]	-1.97	-2.86	-1.07
	Solomon et al. 1997 [32]	-0.04	-0.30	0.21
	Van der Ploeg et al. 2011 [48]	0.20	-0.22	0.62
	Zhang et al. 2006 [36]	-0.36	-0.53	-0.19
	OVERALL	-0.66	-1.32	-0.00
Figure 6	Badon et al. 2014 [39]	-0.64	-1.18	-0.11
	Dempsey et al. 2004 [40]	-1.71	-2.64	-0.77
	Morkrid et al. 2014 [61]	-0.37	-0.70	-0.05
	Solomon et al. 1997 [32]	-0.27	-0.76	0.22
	OVERALL	-0.62	-1.09	-0.14

Figure S1: Assessing the risk of publication bias using funnel plots for different metaanalyses. lnOR, natural log odds ratio

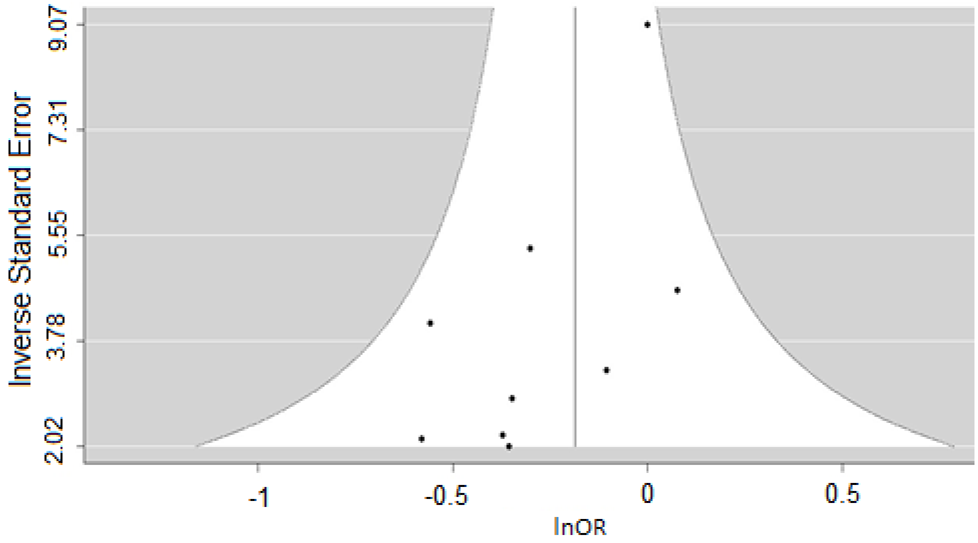

Figure S1a. Any type of PA in early pregnancy versus none (n studies = 9, z = -0.65, p = 0.52).

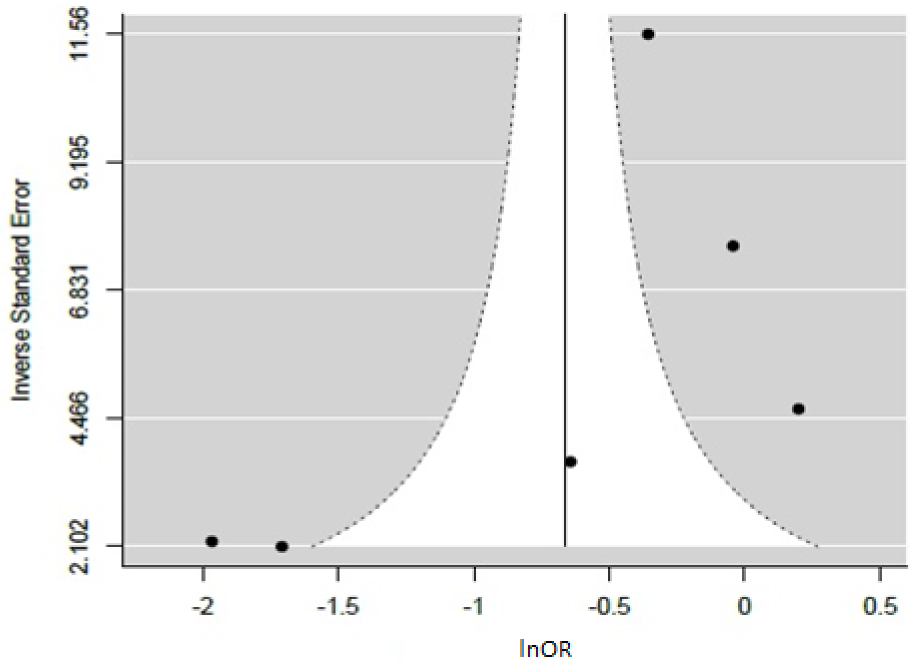

Figure S1b. Pre-pregnancy LTPA high versus none reported in MET.hr/wk (n studies = 6, z = -2.96, p = 0.003).

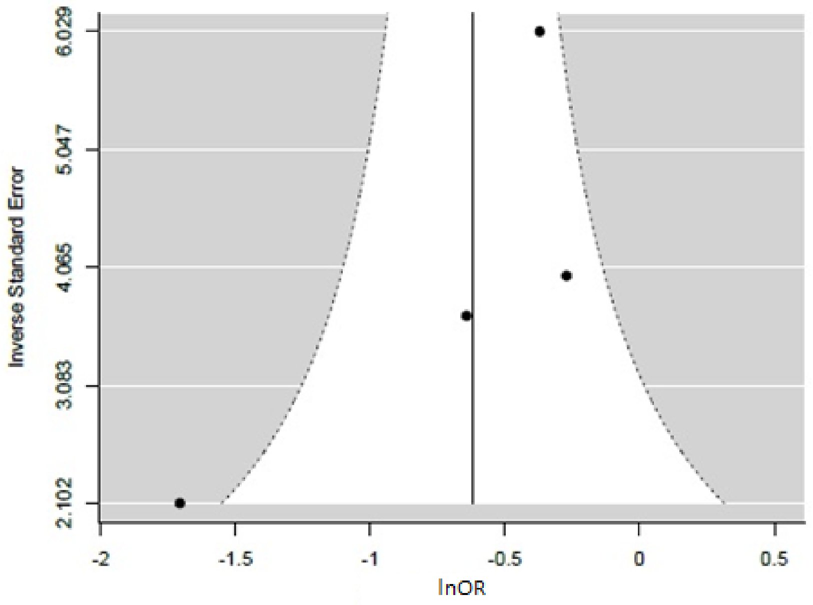

Figure S1c. Pre-pregnancy LTPA high versus none levels reported in hr/wk, (n studies = 4, z = -2.34, p = 0.02). Due to insufficient number of studies reporting on early pregnancy
